# P5CS-coupled proline metabolism manipulates metabolic dysfunction-associated steatotic liver disease

**DOI:** 10.1093/lifemeta/loaf040

**Published:** 2025-11-19

**Authors:** Zan Lyu, Sike Yu, Chang Peng, Wenbiao Wu, Wenhua Yang, Huan Ma, Yan Sun, Liya Jing, Hongyu Gu, Erjiang Tang, Xuemei Zhang, Huihong Jiang, Haowen Jiang, Jia Li

**Affiliations:** Department of Pharmacology, School of Pharmacy, Fudan University, Shanghai 201203, China; State Key Laboratory of Chemical Biology, Shanghai Institute of Materia Medica, Chinese Academy of Sciences, Shanghai 201203, China; Key Laboratory of Glyco-drug Research of Zhejiang Province, School of Pharmaceutical Science and Technology, Hangzhou Institute for Advanced Study, University of Chinese Academy of Sciences, Hangzhou, Zhejiang 310024, China; Key Laboratory of Glyco-drug Research of Zhejiang Province, School of Pharmaceutical Science and Technology, Hangzhou Institute for Advanced Study, University of Chinese Academy of Sciences, Hangzhou, Zhejiang 310024, China; Key Laboratory of Glyco-drug Research of Zhejiang Province, School of Pharmaceutical Science and Technology, Hangzhou Institute for Advanced Study, University of Chinese Academy of Sciences, Hangzhou, Zhejiang 310024, China; School of Life Science and Technology, Shanghai Tech University, Shanghai 201210, China; State Key Laboratory of Chemical Biology, Shanghai Institute of Materia Medica, Chinese Academy of Sciences, Shanghai 201203, China; School of Life Science and Technology, Shanghai Tech University, Shanghai 201210, China; School of Life Science and Technology, Shanghai Tech University, Shanghai 201210, China; School of Life Science and Technology, Shanghai Tech University, Shanghai 201210, China; Department of General Surgery, Yangpu Hospital, School of Medicine, Tongji University, Shanghai 200090, China; Center for Clinical Research and Translational Medicine, Yangpu Hospital, School of Medicine, Tongji University, Shanghai 200090, China; Department of Pharmacology, School of Pharmacy, Fudan University, Shanghai 201203, China; School of Pharmacy, East China Normal University, Shanghai 200241, China; Department of General Surgery, Yangpu Hospital, School of Medicine, Tongji University, Shanghai 200090, China; Center for Clinical Research and Translational Medicine, Yangpu Hospital, School of Medicine, Tongji University, Shanghai 200090, China; State Key Laboratory of Chemical Biology, Shanghai Institute of Materia Medica, Chinese Academy of Sciences, Shanghai 201203, China; State Key Laboratory of Chemical Biology, Shanghai Institute of Materia Medica, Chinese Academy of Sciences, Shanghai 201203, China; Key Laboratory of Glyco-drug Research of Zhejiang Province, School of Pharmaceutical Science and Technology, Hangzhou Institute for Advanced Study, University of Chinese Academy of Sciences, Hangzhou, Zhejiang 310024, China; School of Life Science and Technology, Shanghai Tech University, Shanghai 201210, China; Zhongshan Institute for Drug Discovery, Shanghai Institute of Materia Medica, Chinese Academy of Sciences, Zhongshan Tsuihang New District, Zhongshan, Guangdong 528400, China; Shandong Laboratory of Yantai Drug Discovery, Bohai Rim Advanced Research Institute for Drug Discovery, Yantai, Shandong 264117, China

**Keywords:** MASLD, P5CS, lipid accumulation, mitochondrial dysfunction, proline

## Abstract

Metabolic dysfunction-associated steatotic liver disease (MASLD), characterized by hepatic steatosis, inflammation, and fibrosis, has reached epidemic proportions globally. Emerging evidence highlights a close association between amino acid metabolic dysregulation and MASLD pathogenesis, however, the precise mechanisms remain elusive. In this study, we identify pyrroline-5-carboxylate synthase (P5CS), a pivotal enzyme in proline biosynthesis, as a critical regulator of hepatic proline production and a key driver of MASLD progression. Based on comprehensive analysis of clinical samples from MASLD patients and experimental mouse models, we demonstrate that elevated hepatic and plasma proline levels, resulting from increased P5CS expression, are strongly correlated with disease severity. Genetic overexpression of P5CS in the livers of mice exacerbates diet-induced MASLD, whereas its knockdown exhibits protective profiles. Notably, proline supplementation abolishes the beneficial effects of P5CS knockdown, confirming the causal role of proline overproduction in MASLD pathogenesis. Mechanistically, P5CS-mediated proline accumulation impairs mitochondrial function, thereby disrupting fatty acid oxidation and promoting hepatic lipid accumulation. Pharmacological inhibition of P5CS activity could restore mitochondrial capacity. Thus, our findings establish P5CS-regulated proline metabolism as a novel pathogenic mechanism of MASLD and provide a potential approach for MASLD therapy.

## Introduction

Metabolic dysfunction-associated steatotic liver disease (MASLD), characterized by hepatic steatosis with potential progression to metabolic dysfunction-associated steatohepatitis (MASH), has emerged as a global health crisis [1–4]. MASLD is becoming a leading cause of end-stage liver disease, such as cirrhosis, liver failure, and liver cancer, which requires liver transplantation [5, 6]. As expected, MASLD imposes a major economic burden on society and reduces the health-related quality of life of affected patients [7, 8]. At present, significant research efforts are dedicated to developing treatments for MASLD, but current therapeutic options remain insufficient to meet clinical demands [9–12]. There is an urgent need to identify novel therapeutic targets and develop effective pharmacological interventions for MASLD therapy.

Hepatic steatosis is the initial clinicopathological manifestation of MASLD, which progressively evolves into severe hepatic injury characterized by irreversible inflammatory and fibrotic changes [13–15]. The effects of MASLD to MASH are mediated through mechanisms that include inflammation, oxidative stress, dysbiosis, and predisposition through genetic makeup [16]. Based on clinical and mechanistic evidence, it seems that the intervention of the development of hepatic steatosis represents a crucial therapeutic strategy for MASLD therapy [16–18]. At the current stage, factors that cause and exacerbate steatosis development and trigger hepatic inflammatory responses and fibrogenesis may be at play in a parallel or sequential manner and with different hierarchies along the whole spectrum of the disease [19, 20]. The pathogenesis of disturbed hepatic lipid homeostasis results from both intrahepatic metabolic dysregulation and extrahepatic pathological factors [19]. In hepatocytes, the dysfunction of lipid trafficking, including increased lipid uptake [21], decreased very low-density lipoprotein secretion [22, 23], enhanced *de novo* lipogenesis [24], and impaired hepatic catabolic metabolism [25, 26], collectively reprograms lipid metabolism and promotes steatosis. On the other hand, extrahepatic factors such as gut dysbiosis–mediated inflammatory cytokine secretion [27, 28] and adipocyte expansion–derived adipokine release further exacerbate hepatic lipid deposition [29–31]. Thus, the complex and multifactorial pathogenesis of MASLD poses significant challenges in identifying pharmacological targets.

Emerging studies indicate that intrahepatic lipid accumulation in MASLD involves more than just lipid metabolic dysfunction, with perturbations in amino acid metabolism [32, 33]. Previous studies have demonstrated that the highly active glutaminase 1 (GLS1) isoform disrupts glutamine metabolism, impairing its cataplerotic contribution to mitochondrial metabolism while simultaneously reducing oxidative stress defenses, ultimately promoting lipid accumulation and MASLD progression [34]. Furthermore, increased hepatic L-aspartate transported from the gut can ameliorate MASLD by activating liver kinase B1 (LKB1)-AMP activated protein kinase (AMPK) signaling to induce fatty acid oxidation [32]. In addition, serum metabolomics-based profiles of MASLD patients further illustrate the importance of amino acids such as arginine, proline, and histidine in MASLD pathogenesis [35]. These findings not only establish a significant association between dysregulated amino acid metabolism and MASLD progression but also provide a potential approach for MASLD therapy.

Proline, one of the 12 non-essential amino acids in humans, is a small, cyclic, nonpolar, nontoxic, odorless, and sweet-tasting amino acid [36]. In cell biology, proline has multifaceted roles such as: (1) a main source of extracellular collagen biosynthesis; (2) a modulator of cellular signaling pathways including the amino acid stress response and the extracellular signal-regulated kinase pathway; (3) a participant in maintaining cellular redox balance; (4) and an inducer of the proliferation of stem and tumor cells [36–38]. As was previously reported, type 2 diabetes (T2DM) has been linked to higher plasma and liver proline levels. Proline enhances gluconeogenesis and hyperglycemia via diminishing hepatocyte paraspeckles [39]. Moreover, increased plasma proline concentrations involved in aberrated amino acid levels is observed in patients and MASH mouse models [40–43]. However, the linkage and the underlying mechanisms between proline levels and MASLD are unclarified. Herein, we clarify the linkage between proline levels and MASLD progression. We identified that pyrroline-5-carboxylate synthetase (P5CS; encoded by aldehyde dehydrogenase family 18 member A1 (*ALDH18A1*) gene), a key enzyme in proline biosynthesis, was significantly upregulated in the livers of clinical patients with higher liver injury and in the livers of MASH mouse models. As expected, the upregulation of P5CS in pathological conditions increased both the hepatic and plasma proline levels. P5CS inhi­bited mitochondrial function in hepatocytes via its downstream enzyme product proline, which exacerbated liver lipid accumulation and MASLD progression. These findings collectively illustrate the significant regulatory function of P5CS in the remodeling of proline metabolism and suggest it as a potential target for future therapeutic interventions in MASLD.

## Results

### P5CS (*ALDH18A1*) expression is upregulated during the progression of MASLD

**Figure 1:**
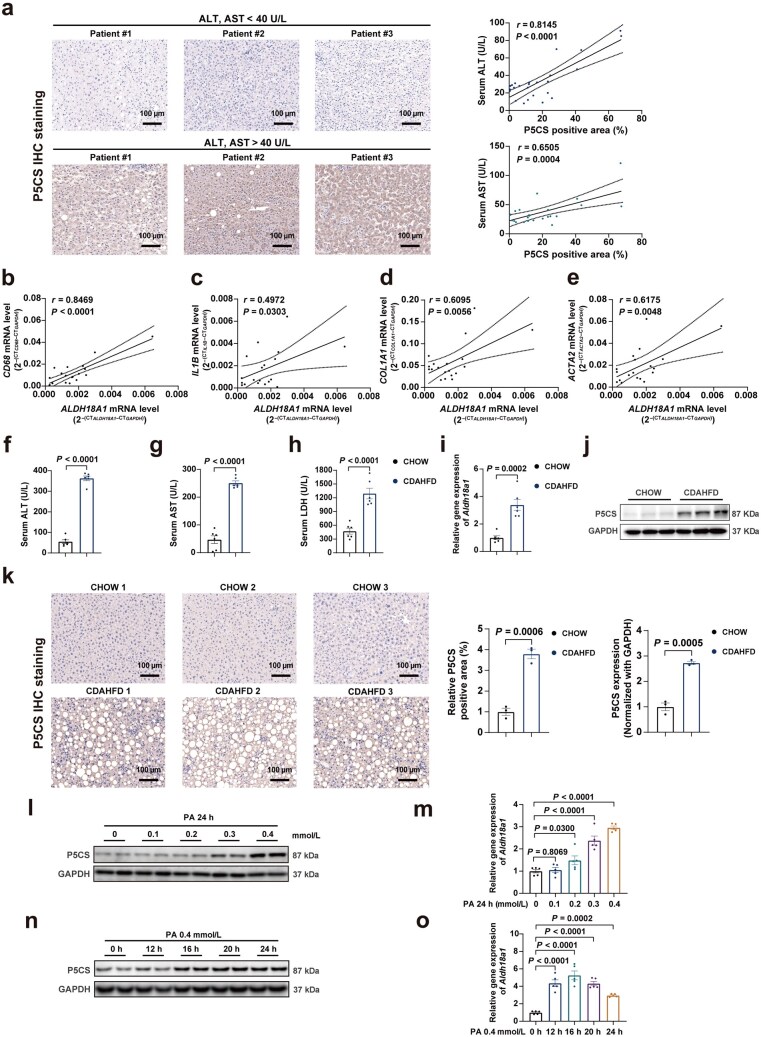
P5CS (*ALDH18A1*) expression is upregulated during the progression of MASLD. (a) IHC staining of hepatic P5CS in clinical patients (*n *= 25), and the correlation between P5CS positive area and serum ALT and AST contents. Scale bar, 100 µm. (b–e) Pearson *r* and *P* values for the correlation of *ALDH18A1* mRNA expression level with mRNA expression levels of inflammatory genes and fibrogenesis-related genes (*n *= 19). (f–h) Serum ALT (f), AST (g), and LDH (h) levels of CHOW- or CDAHFD-fed mice (*n *= 6 mice/group). (i) Relative mRNA level of *Aldh18a1* in the livers of CHOW- or CDAHFD-fed mice (*n *= 6 mice/group). (j) Immunoblot analysis (upper) and quantification (bottom) of P5CS expression in the livers of CHOW- or CDAHFD-fed mice (*n *= 3 mice/group). Protein expression was normalized to GAPDH level. (k) IHC staining (left) and quantification (right) of hepatic P5CS expression in the livers of CHOW- or CDAHFD-fed mice (*n *= 3 mice/group). (l and m) P5CS protein (l) and *Aldh18a1* mRNA (m) expression levels of AML12 cells treated with PA at different concentrations for 24 h. (n and o) P5CS protein (n) and *Aldh18a1* mRNA (o) levels in AML12 cells treated with 0.4 mmol/L PA for the indicated time periods. Data are shown as mean ± SEM. Two-tailed Student’s *t*-test was used for two-group comparisons, and one-way ANOVA was used for multi-group comparisons. IHC positive area was quantified by ImageJ software. The correlation analysis was performed using Spearman correlation anatlysis. ALT, alanine aminotransferase; AST, aspartate aminotransferase; CDAHFD, choline-deficient, L-amino acid-defined, high-fat diet; LDH, lactate dehydrogenase; PA, palmitic acid.

To examine the association between P5CS (*ALDH18A1*) and MASLD, the authors first collected the plasma and liver tissues of patients to detect the association between P5CS and MASLD. Immunohistochemistry (IHC) results showed that the plasma levels of alanine aminotransferase (ALT; normal level is 0–40 U/L) and aspartate aminotransferase (AST; normal level is 0–40 U/L), the markers of liver injury, were positively correlated with liver P5CS expression (Fig. 1a). Consistently, immunoblotting results also indicated the elevation of P5CS in the liver tissues from patients with higher plasma ALT and AST levels (Supplementary Fig. S1a). Furthermore, hepatic *ALDH18A1* mRNA level exhibited a positive correlation with the expression of proinflammatory and fibrotic genes, such as cluster of differentiation 68 (*CD68*), interleukin-1beta (*IL1B*), collagen type I alpha 1 chain (*COL1A1*), and smooth muscle alpha-actin 2 (*ACTA2*) (Fig. 1b–e). The authors also analyzed the *ALDH18A1* expression in the transcriptome data of liver tissues from MASLD patients by analyzing the Gene Expression Omnibus (GEO) public database. In comparison to healthy individuals, hepatic *ALDH18A1* expression was significantly elevated in MASH and further increased with the advancement of the MASLD stage (­Supplementary Fig. S1b–d). The changes in P5CS (*Aldh18a1*) expression in MASH liver tissues were then investigated in two mouse models of MASH induced by a choline-deficient, L-amino acid-defined, high-fat diet (CDAHFD) and a high-fat/high-cholesterol, high carbohydrate (HFHC) diet, respectively. In both MASH mouse models, plasma liver injury parameters were considerably elevated (Fig. 1f–h; ­Supplementary Fig. S1e–g). Moreover, hepatic *Aldh18a1* mRNA expression increased markedly (Fig. 1i; Supplementary Fig. S1h), verified by immunoblotting and IHC results (Fig. 1j and k; ­Supplementary Fig. S1i and j). In addition, *Aldh18a1* was also found to be upregulated in liver tissues of other MASH mouse models, according to the findings from three GEO datasets (Supplementary Fig. S1k–m). As widely known, lipotoxicity is the main cause for hepatic steatosis and MASLD development. Then, we cultured cells with fatty acids. Consistent with the aforementioned *in vivo* findings, *in vitro* experiments indicated that P5CS (*Aldh18a1*) expression increased in a concentration-dependent and time-dependent manner in alpha mouse liver 12 (AML12) cells after exposure to palmitic acid (PA; Fig. 1l–o). Therefore, these results suggest that the increment of P5CS (*ALDH18A1*) might contribute to MASLD development.

### Liver-specific overexpression of P5CS exacerbates CDAHFD-induced MASH

**Figure 2:**
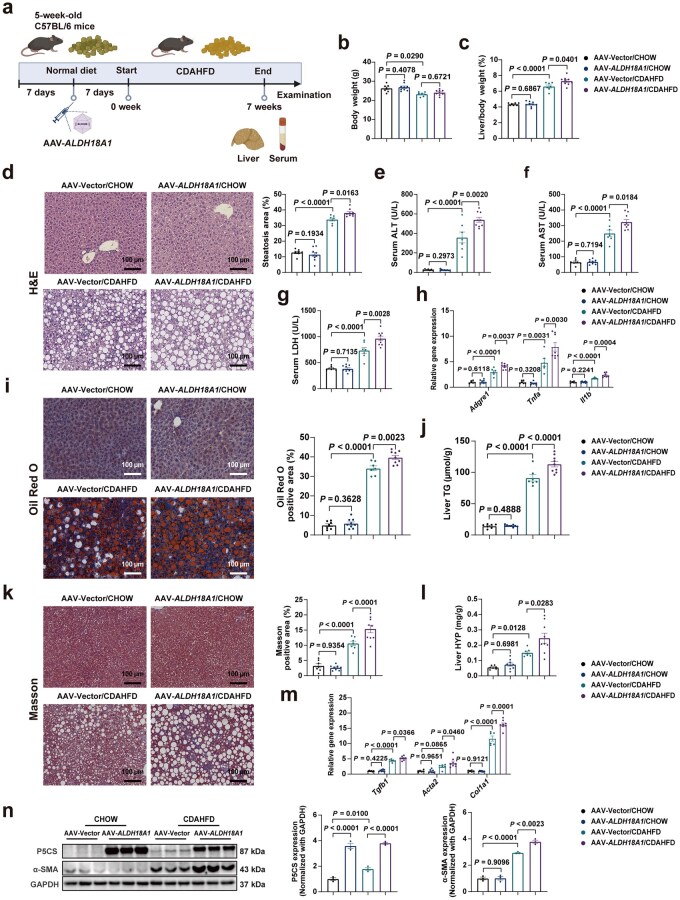
Liver-specific overexpression of P5CS exacerbates the CDAHFD-induced MASH. (a) Schematic diagram of experiment design for P5CS overexpression and CDAHFD-induced MASH mouse model. Mice injected with AAV-Vector and AAV-*ALDH18A1* were fed with CHOW diet or CDAHFD for 7 weeks (*n *= 7–9 mice/group). (b) Body weight of mice in the indicated groups (*n *= 7–9 mice/group). (c) Liver/body weight ratio of mice in the indicated groups (*n *= 7–9 mice/group). (d) H&E staining (left) and steatosis area quantification (right) of liver sections from indicated groups of mice (*n *= 7–9 mice/group). Scale bar, 100 µm. (e–g) Serum ALT (e), AST (f), and LDH (g) levels of mice in the indicated groups (*n *= 7–9 mice/group). (h) Relative mRNA levels of hepatic proinflammatory genes in the indicated groups of mice (*n *= 6–8 mice/group). (i) Oil Red O staining (left) and quantification (right) of liver sections from indicated groups of mice (*n *= 7–9 mice/group). Scale bar, 100 µm. (j) Hepatic TG content of mice in the indicated groups (*n *= 7–9 mice/group). (k) Masson staining (left) and quantification (right) of liver sections from indicated groups of mice (*n *= 7–9 mice/group). Scale bar, 100 µm. (l) Hepatic HYP content of mice in the indicated groups (*n *= 7–9 mice/group). (m) Relative mRNA levels of hepatic fibrogenesis-related genes in the indicated groups of mice (*n *= 6–8 mice/group). (n) Immunoblot analysis (left) and quantification (right) of P5CS and α-SMA levels in the livers of mice in the indicated groups (*n *= 3 mice/group). Protein expression was normalized to GAPDH level. Data are shown as mean ± SEM. Two-way ANOVA was used for multi-group comparisons. The steatosis area, Oil Red O positive area, and Masson positive area were quantified by ImageJ software. ALT, alanine aminotransferase; AST, aspartate aminotransferase; CDAHFD, choline-deficient, L-amino acid-defined, high-fat diet; HYP, hydroxyproline; LDH, lactate dehydrogenase; TG, triglyceride.

To further investigate the effects of P5CS on MASLD/MASH progression, the authors generated an *ALDH18A1* expression vector utilizing adeno-associated virus (AAV) type 8 (AAV-*ALDH18A1*). Immunoblotting experiments demonstrated that tail-vein injection of AAV-*ALDH18A1* effectively increased P5CS expression in the liver, while not affecting other tissues (Supplementary Fig. S2a). Mice were given AAV-*ALDH18A1* injections and subjected to CDAHFD feeding for 7 weeks before being sacrificed (Fig. 2a). In comparison with the control group, mice with liver-specific P5CS overexpression had an elevated liver/body weight ratio under CDAHFD feeding conditions, with no difference in body weight (Fig. 2b and c). Furthermore, hematoxylin and eosin (H&E) analysis demonstrated that liver P5CS overexpression in CDAHFD-MASH mice markedly increased the liver steatosis area (Fig. 2d), together with higher plasma liver injury markers ALT, AST, and lactate dehydrogenase (LDH; Fig. 2e–g). In addition, consistent with the upward trend of liver damage parameters in the serum of liver P5CS-overexpressed CDAHFD-MASH mice, the overexpression of P5CS in primary hepatocytes significantly elevated PA-induced lipotoxicity, resulting in increased liver damage indicators in cell supernatants (­Supplementary Fig. S2b–e). Mice with liver P5CS overexpression also exhibited greater inflammatory responses following CDAHFD consumption, as evidenced by the higher mRNA expression levels of proinflammatory genes in the liver (Fig. 2h). Oil Red O and Masson staining revealed that the overexpression of liver P5CS significantly promoted CDAHFD-induced hepatic lipid accumulation and fibrosis, as evidenced by elevated hepatic triglyceride (TG) and hydroxyproline (HYP) contents (Fig. 2i–l). Moreover, reverse transcription quantitative real-time PCR (RT-qPCR) and immunoblotting analyses indicated increased expression of fibrosis markers in the livers of P5CS-overexpressed CDAHFD-MASH mice (Fig. 2m and n). These data demonstrate that P5CS promotes CDAHFD-induced MASH progression.

### Liver-specific P5CS knockdown alleviates CDAHFD-induced MASH

**Figure 3:**
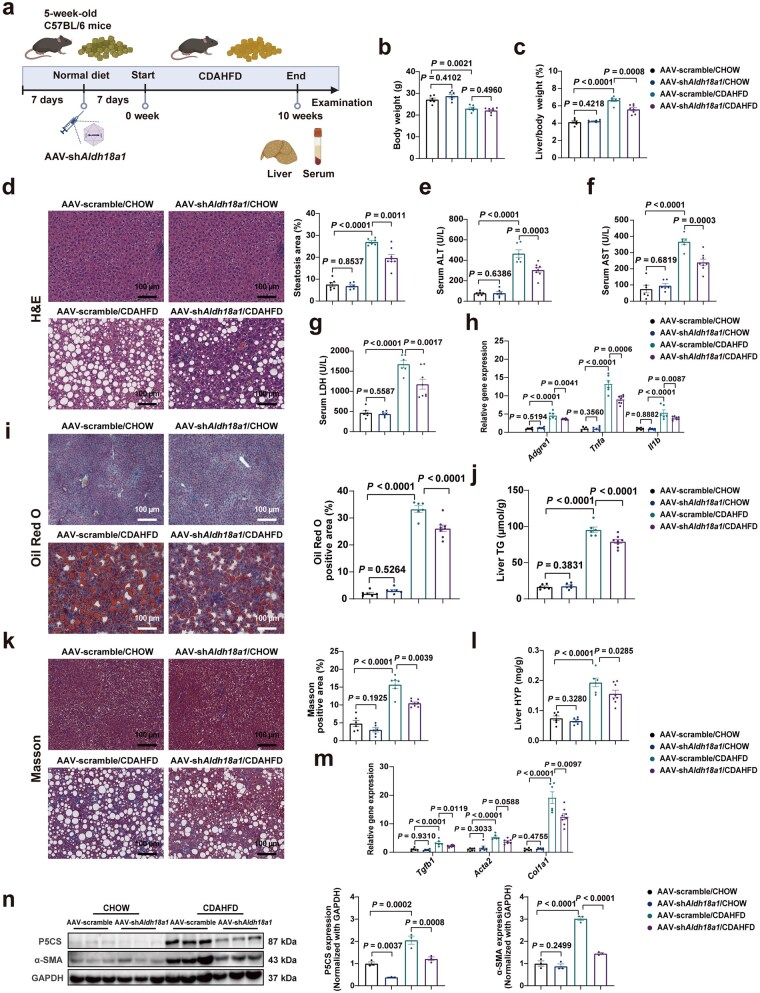
Liver-specific knockdown of P5CS alleviates CDAHFD-induced MASH. (a) Schematic diagram of experiment design for P5CS knockdown and CDAHFD-induced MASH mouse model. Mice injected with AAV-scramble and AAV-sh*Aldh18a1* were fed with CHOW diet or CDAHFD for 10 weeks (*n *= 6–8 mice/group). (b) Body weight of mice in the indicated groups (*n *= 6–8 mice/group). (c) Liver/body weight ratio of mice in the indicated groups (*n *= 6–8 mice/group). (d) H&E staining (left) and steatosis area quantification (right) of liver sections from the indicated groups of mice (*n *= 6–8 mice/group). Scale bar, 100 µm. (e–g) Serum ALT (e), AST (f), and LDH (g) levels in the indicated groups of mice (*n *= 6–8 mice/group). (h) Relative mRNA levels of hepatic proinflammatory genes in the indicated groups of mice (*n *= 5–8 mice/group). (i) Oil Red O staining (left) and quantification (right) of liver sections from the indicated groups of mice (*n *= 6–8 mice/group). Scale bar, 100 µm. (j) Hepatic TG content of mice in the indicated groups (*n *= 6–8 mice/group). (k) Masson staining (left) and quantification (right) of liver sections from the indicated groups of mice (*n *= 6–8 mice/group). Scale bar, 100 µm. (l) Hepatic HYP content of mice in the indicated groups (*n *= 6–8 mice/group). (m) Relative mRNA levels of hepatic fibrogenesis-related genes in the indicated groups of mice (*n *= 6–8 mice/group). (n) Immunoblot analysis (left) and quantification (right) of P5CS and α-SMA levels in the livers of mice in the indicated groups (*n *= 3 mice/group). Protein expression was normalized to GAPDH level. Data are shown as mean ± SEM. Two-way ANOVA was used for multi-group comparisons. The steatosis area, Oil Red O positive area, and Masson positive area were quantified by ImageJ software. ALT, alanine aminotransferase; AST, aspartate aminotransferase; CDAHFD, choline-deficient, L-amino acid-defined, high-fat diet; HYP, hydroxyproline; LDH, lactate dehydrogenase; TG, triglyceride.

In parallel, the authors also generated an *Aldh18a1* shRNA using AAV type 8 (AAV-sh*Aldh18a1*) to specifically knock down P5CS in the liver (Supplementary Fig. S3a). The experiment design is shown in Fig. 3a. The knockdown of liver P5CS did not affect body weight but reduced the liver/body weight ratio under CDAHFD feeding conditions (Fig. 3b and c). H&E staining analysis indicated that CDAHFD-induced hepatic steatosis was ameliorated in the livers of P5CS-knockdown mice (Fig. 3d). The decreased serum levels of ALT, AST, and LDH demonstrated a significant improvement in liver injury in mice with liver P5CS knockdown after CDAHFD consumption (Fig. 3e–g). Furthermore, PA-induced lipotoxicity in primary hepatocytes was diminished after P5CS knockdown, as indicated by reduced cell damage (Supplementary Fig. S3b–e). Decreased mRNA expression levels of proinflammatory genes proved that mice with liver P5CS knockdown exhibited attenuated inflammatory responses to CDAHFD consumption (Fig. 3h). Oil Red O and Masson staining analyses demonstrated that the knockdown of liver P5CS in CDAHFD-MASH mice improved hepatic lipid accumulation and fibrosis, which is consistent with diminished hepatic TG and HYP contents (Fig. 3i–l). In addition, the expression levels of liver fibrogenesis-related genes and alpha-smooth muscle actin (α-SMA) protein were reduced in the livers of P5CS-knockdown CDAHFD-MASH mice, indicating the alleviation of hepatic fibrosis (Fig. 3m and n). These findings suggest that the suppression of P5CS could facilitate the mitigation of MASH progression.

### P5CS promotes lipid accumulation via the inhibition of mitochondrial function in hepatocytes

As previously demonstrated by *in vivo* and *in vitro* studies, our ­findings have established the indispensable role of P5CS in the development of MASLD, particularly in regulating hepatic lipid metabolism. These indicated that P5CS might have an unrecognized role in the cellular lipid accumulation driven by metabolic reprogramming in hepatocytes. Because many existing MASH mouse models induced by diet, genetics, and toxins caused hepatic metabolic reprogramming, the authors then examined how the CDAHFD used in this study affected hepatic lipid metabolism and how P5CS thereby promoted hepatic lipid accumulation in CDAHFD-MASH mice. The authors thus conducted RNA sequencing (RNA-seq) on total RNA extracted from the livers of mice fed with CDAHFD or normal diet (Supplementary Fig. S4a) for gene analysis (Supplementary Fig. S4b). Gene Ontology (GO) and Kyoto Encyclopedia of Genes and Genomes (KEGG) pathway enrichment analyses indicated that the lipid metabolic process was significantly impaired in the livers of CDAHFD-MASH mice (Supplementary Fig. S4c and d). Moreover, gene set enrichment analysis confirmed a downregulation in fatty acid beta-oxidation and mitochondrial function pathways in CDAHFD livers, while fatty acid biosynthetic process was not affected (Supplementary Fig. S4e–j).

**Figure 4:**
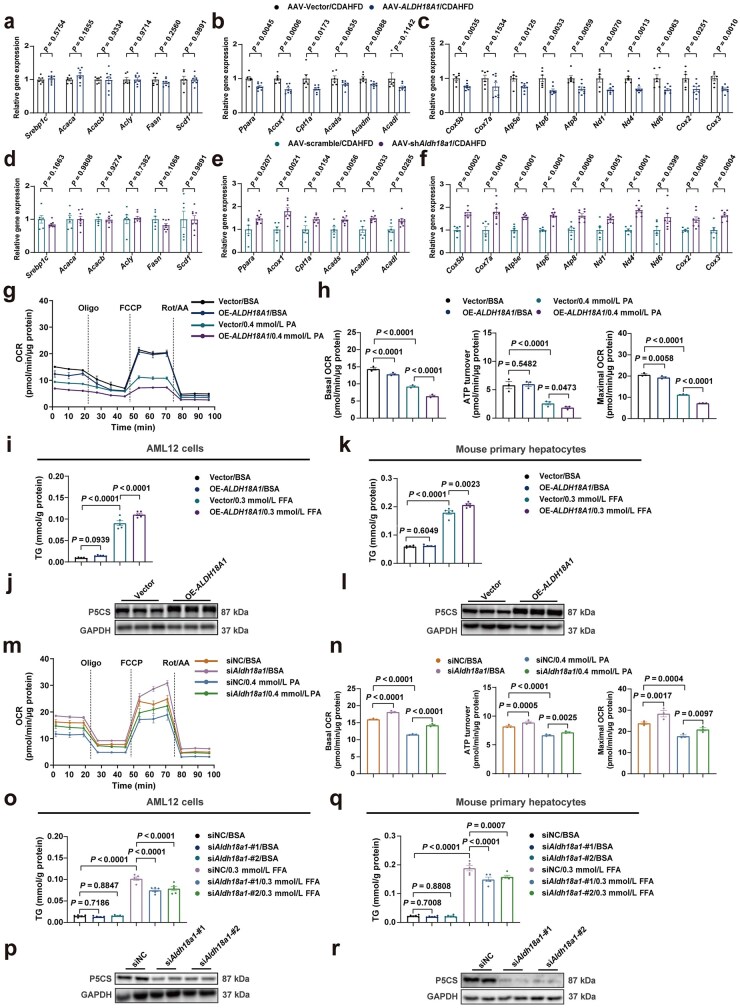
P5CS promotes lipid accumulation via the inhibition of mitochondrial function in hepatocytes. (a–c) Relative mRNA levels of genes related to fatty acid biosynthesis (a), fatty acid oxidation (b), and mitochondrial function (c) in the livers of CDAHFD-fed Vector-overexpressed and P5CS-overexpressed mice (*n *= 6–8 mice/group). (d–f) Relative mRNA levels of genes related to fatty acid biosynthesis (d), fatty acid oxidation (e), and mitochondrial function (f) in the livers of CDAHFD-fed negative-control and P5CS-knockdown mice (*n *= 6 − 8 mice/group). (g) Cellular respiration of Vector-overexpressed and P5CS-overexpressed AML12 cells treated with BSA or PA. (h) Basal, ATP-linked, and maximal OCRs of (g) measured by seahorse analysis (*n *= 4). (i) Cellular TG content of Vector-overexpressed and P5CS-overexpressed AML12 cells followed by treatment with BSA or FFA (*n *= 5). (j) P5CS expression level in AML12 cells transfected with vector or *ALDH18A1* plasmid. (k) Cellular TG content of Vector-overexpressed and P5CS-overexpressed primary hepatocytes followed by treatment with BSA or FFA (*n *= 5). (l) P5CS expression level in primary hepatocytes transfected with Vector or *ALDH18A1* plasmid. (m) Cellular respiration of negative-control and P5CS-knockdown AML12 cells treated with BSA or PA. (n) Basal, ATP-linked, and maximal OCRs of (m) measured by seahorse analysis (*n *= 4). (o) Cellular TG content of negative-control and P5CS-knockdown AML12 cells followed by treatment with BSA or FFA (*n *= 5). (p) P5CS expression level in AML12 cells transfected with negative control siRNA or si*Aldh18a1*. (q) Cellular TG content of negative-control and P5CS-knockdown primary hepatocytes followed by treatment with BSA or FFA (*n *= 5). (r) P5CS expression level in primary hepatocytes transfected with negative control siRNA or si*Aldh18a1*. Data are shown as mean ± SEM. Two-tailed Student’s *t*-test was used for two-group comparisons, and two-way ANOVA was used for multi-group comparisons. FFA, free fatty acid; OCR, oxygen consumption rate; PA, palmitic acid; TG, triglyceride.

Next, the authors investigated whether the role of P5CS in modulating MASH progression resulted from the alterations in fatty acid beta-oxidation and mitochondrial function. RT-qPCR analysis revealed that P5CS overexpression reduced the hepatic mRNA levels of genes related to fatty acid oxidation and mitochondrial function, whereas genes involved in fatty acid biosynthesis remained unchanged (Fig. 4a–c). Conversely, P5CS knockdown elevated hepatic mRNA levels of genes involved in fatty acid oxidation and mitochondrial function, but genes implicated in fatty acid biosynthesis were unaffected (Fig. 4d–f). To further determine whether P5CS modulated lipid metabolism by affecting hepatocyte mitochondrial function, cellular respiration assay was conducted on AML12 cells. In P5CS-overexpressed cells, mitochondrial dysfunction induced by PA treatment was significantly exacerbated (Fig. 4g and h). In addition, the free fatty acid (FFA)-induced TG accumulation in hepatocytes was significantly increased in the P5CS-overexpression group, as observed in both AML12 cells and primary hepatocytes (Fig. 4i–l). In contrast, markedly less PA-induced mitochondrial dysfunction occurred in the P5CS-knockdown AML12 cells (Fig. 4m and n). Furthermore, in both AML12 cells and primary hepatocytes, FFA-induced TG accumulation was significantly reduced in the P5CS-knockdown group (Fig. 4o–r). Except for the changes of fatty acid oxidation-related genes, to determine whether fatty acid oxidation was directly altered by P5CS, a metabolic flux analysis was conducted through stable isotope tracing and changes in the levels of mitochondrial tricarboxylic acid (TCA) cycle metabolites derived from PA were observed (Supplementary Fig. S5a). The results indicated that after P5CS knockdown and treatment with [U–^13^C_16_]-PA, the levels of ­cellular TCA cycle-related metabolites derived from [U–^13^C_16_]-PA were significantly increased in comparison to the control group (Supplementary Fig. S5b), meaning that the inhibition of P5CS enhances the activities of cellular TCA cycle and fatty acid oxidation. Taken together, our data indicate that P5CS is crucial in the progression of MASLD by disrupting mitochondrial function and lipid metabolism in hepatocytes.

### P5CS-coupled proline metabolism is disrupted during MASLD progression

**Figure 5:**
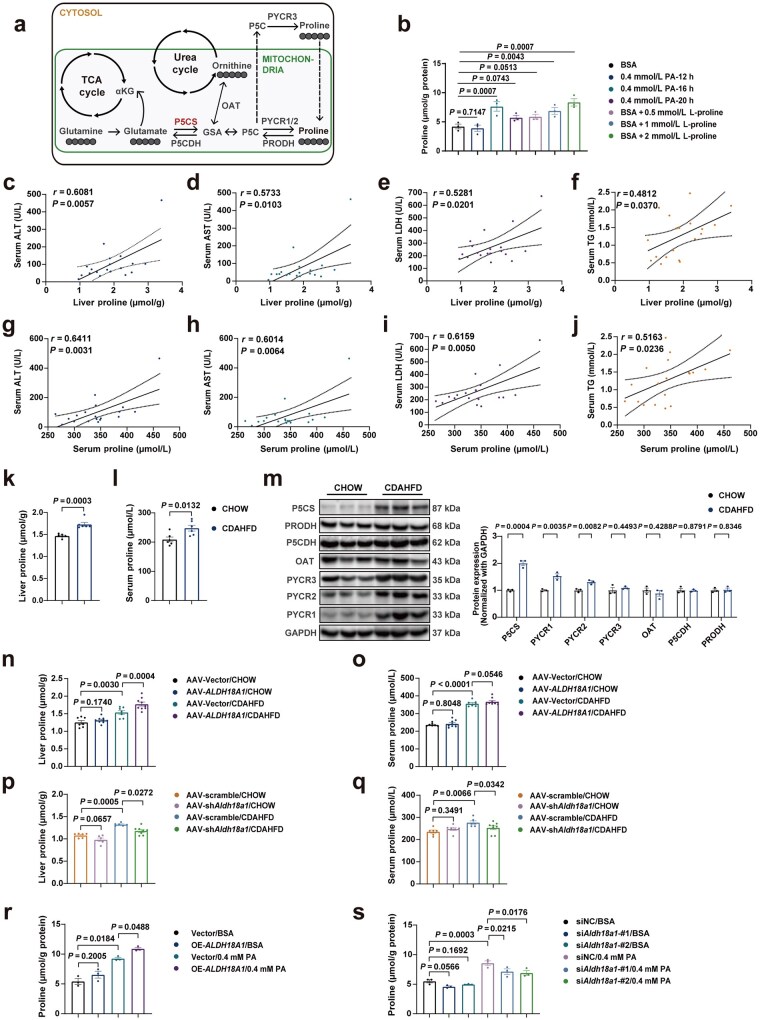
P5CS-coupled proline metabolism is disrupted during MASLD progression. (a) Summary of key enzymes involved in proline metabolism. (b) ­Intracellular proline level in AML12 cells treated with PA or L-proline (*n *= 3). (c–f) Correlations between liver proline content and serum ALT (c), AST (d), LDH (e), and TG (f) levels in clinical patients (*n *= 19). (g–j) Correlation between serum proline content and serum ALT (g), AST (h), LDH (i), and TG (j) levels in clinical patients (*n *= 19). (k) Liver proline content of CHOW- or CDAHFD-fed mice (*n *= 6 mice/group). (l) Serum proline content of CHOW- or CDAHFD-fed mice (*n *= 6 mice/group). (m) Immunoblot analysis (left) and quantification (right) of the levels of proline metabolism-related proteins in the livers of CHOW- or CDAHFD-fed mice (*n *= 3 mice/group). Protein expression was normalized to GAPDH level. (n) Liver proline content of CHOW- or CDAHFD-fed liver-specific Vector-overexpressed and liver-P5CS-overexpressed mice (*n *= 7–9 mice/group). (o) Serum proline content of CHOW- or CDAHFD-fed liver-specific Vector-overexpressed and liver-P5CS-overexpressed mice (*n *= 7–9 mice/group). (p) Liver proline content of CHOW- or CDAHFD-fed negative-control and liver-specific P5CS-knockdown mice (*n *= 6–8 mice/group). (q) Serum proline content of CHOW- or CDAHFD-fed negative-control and liver-specific P5CS-knockdown mice (*n *= 6–8 mice/group). (r) Intracellular proline content of Vector- and P5CS-overexpressed AML12 cells treated with BSA or PA (*n *= 3). (s) Intracellular proline content of negative-control and P5CS-knockdown AML12 cells treated with BSA or PA (*n *= 3). Data are shown as mean ± SEM. Two-tailed Student’s *t*-test was used for two-group comparisons. One-way ANOVA (b) and two-way ANOVA (n–s) were used for multi-group ­comparisons. The correlation analysis was performed using Spearman correlation analysis. ALT, alanine aminotransferase; AST, aspartate aminotransferase; CDAHFD, choline-deficient, L-amino acid-defined, high-fat diet; LDH, lactate dehydrogenase; PA, palmitic acid, TG, triglyceride.

It is known that P5CS is the rate-limiting enzyme in the proline biosynthesis pathway (Fig. 5a). PA treatment not only upregulated P5CS expression (Fig. 1l–o) but also induced a significant accumulation of intracellular proline in AML12 cells, comparable to the effects of exogenous L-proline addition (Fig. 5b). We subsequently investigated whether proline metabolism was influenced by the upregulation of P5CS during MASLD progression. Analysis of the GEO public database demonstrated that the livers of MASLD patients had significantly altered expression levels of proline metabolism-related genes (Supplementary Fig. S6a–c). Moreover, correlation analysis revealed that the concentrations of the liver (Fig. 5c–f) and serum proline (Fig. 5g–j) in the clinical patients were positively correlated with plasma levels of ALT, AST, LDH, and TG. In addition, elevated liver and serum proline levels were observed in both the CDAHFD-induced (Fig. 5k and l) and HFHC-induced (Supplementary Fig. S6d and e) MASH mouse models. These data demonstrated that the progression of MASLD was highly linked with the increased liver and serum proline levels. Next, the correlated gene and protein expression levels were determined in the livers of CDAHFD- and HFHC-induced MASH mice. The results indicated that the expression levels of proline biosynthesis-related proteins, P5CS, PYCR1, and PYCR2, were significantly elevated in the livers of CDAHFD-induced MASH mice, while the expression levels of the other two proline biosynthesis-related proteins, PYCR3 and OAT, were unchanged compared to the control group. Moreover, the gene expression levels of hepatic proline catabolism-related proteins P5CDH (encoded by *Aldh4a1* gene) and PRODH exhibited a down-regulation trend in CDAHFD-induced MASH mice; however, their protein expression levels remained unchanged (Fig. 5m; ­Supplementary Fig. S6g). In the livers of HFHC-induced MASH mice, among the proline biosynthesis-related proteins, only P5CS increased at both gene and protein levels. The genes *Aldh4a1* and *Prodh* showed a similar trend of downregulation in the HFHC-induced MASH livers, whereas their protein expression levels remained unchanged (Supplementary Fig. S6f and h). The consistent upregulation of P5CS in both MASH mouse models suggested that it may be a key factor contributing to the proline metabolic disorder.

To further confirm the cellular role of P5CS relative to other proline metabolism-related proteins, AML12 cells were treated with 0.4 mmol/L PA for 16 h, which is consistent with the conditions that the P5CS upregulation and intracellular proline accumulation can be induced. Except for P5CS, the other related proteins did not exhibit any changes or consistent alterations at gene and protein levels (Supplementary Fig. S6i and j), meaning that the primary factor responsible for the elevated level of proline in hepatocytes under MASLD pathological conditions is the upregulation of P5CS. Subsequently, the liver and serum proline concentrations in CDAHFD-induced MASH mice with liver-specific P5CS overexpression and knockdown were measured. The overexpression of P5CS elevated CDAHFD-induced liver proline accumulation, while the serum proline level was not significantly affected (Fig. 5n and o). Conversely, the P5CS knockdown reduced CDAHFD-induced liver and serum proline elevation (Fig. 5p and q). Similar results were observed at the cellular level. P5CS overexpression increased PA-induced intracellular proline accumulation, whereas P5CS knockdown diminished this phenotype (Fig. 5r and s; ­Supplementary Fig. S6k and l). Therefore, these results demonstrate that P5CS might be the core factor disrupting liver proline ­metabolism during MASLD progression.

### P5CS promotes the progression of MASLD depending on its enzyme activity

Based on the aforementioned results, proline has the possibility to serve as the principal downstream factor of P5CS in promoting MASLD progression. To validate this hypothesis, L-proline supplementation was performed in cells with P5CS knockdown. Cellular respiration assays revealed that the knockdown of P5CS improved mitochondrial function, whereas the addition of L-proline removed this effect (Supplementary Fig. S7a and b). Moreover, mitochondrial dysfunction caused by exposure to PA was exacerbated by L-proline addition. The PA-induced mitochondrial dysfunction was improved in the P5CS-knockdown group, but this beneficial effect was blocked by L-proline supplementation (Supplementary Fig. S7c and d). In addition, in both the AML12 cells and primary hepatocytes treated by FFA, the addition of L-proline exacerbated TG accumulation. While TG accumulation was markedly diminished in P5CS-knockdown group, this alleviative effect was also blocked by the addition of exogenous L-proline (Supplementary Fig. S7e−h).

P5CS is a bifunctional enzyme exhibiting glutamate kinase (GK) and gamma-glutamyl phosphate reductase (GPR) activities [44]. To test whether the enzyme activity was required for the function of P5CS, we mutated three main residues (Lys76, Asp247, and Lys311) in the activity center of the GK moiety and the catalytic cysteine (Cys612) of the GPR moiety (Supplementary Fig. S8a). Compared with the overexpression of WT P5CS, the mutant P5CS overexpression did not further increase PA-induced intracellular proline accumulation (Supplementary Fig. S8b). Under bovine serum albumin (BSA) treatment conditions, the overexpression of WT P5CS impaired cellular mitochondrial function, while the overexpression of mutant P5CS did not (Supplementary Fig. S8c and d). When exposed to PA, the overexpression of WT P5CS or mutant P5CS reduced the cellular basal respiration, whereas only the cells overexpressing WT P5CS exhibited impaired cellular ATP production and maximal respiration (Supplementary Fig. S8e and f). Furthermore, in comparison to the overexpression of WT P5CS, the overexpression of mutant P5CS did not significantly increase FFA-induced TG accumulation in AML12 cells and primary hepatocytes (Supplementary Fig. S8g–j).

*In vitro* experimental findings have validated that the enzyme activities of P5CS are essential to its function. P5CS promotes the aggravation of mitochondrial dysfunction and TG accumulation in hepatocytes via its downstream enzyme product, proline. Next, the authors administered L-proline to mice fed with normal diet via daily intraperitoneal injection to observe the effects of proline on normal mice (Supplementary Fig. S9a). After proline intraperitoneal administration, no notable alterations were observed in body weight or liver/body weight ratio (Supplementary Fig. S9b and c), and there was no change in plasma concentrations of liver injury indicators (Supplementary Fig. S9d–f). Compared to the control group and the 0.1 g/kg dosage group, the liver TG contents of mice from the 0.3 g/kg dosage group exhibited a slight upward trend (Supplementary Fig. S9g). Correspondingly, in the 0.3 g/kg dosage group, some of the genes related to liver fatty acid oxidation (*Acadm* and *Acadl*) exhi­bited a slight decrease trend, whereas genes associated with fatty acid synthesis remained unchanged (Supplementary Fig. S9h and i). Furthermore, proline administration did not induce liver inflammation and fibrosis in normal mice (Supplementary Fig. S9j−l).

**Figure 6:**
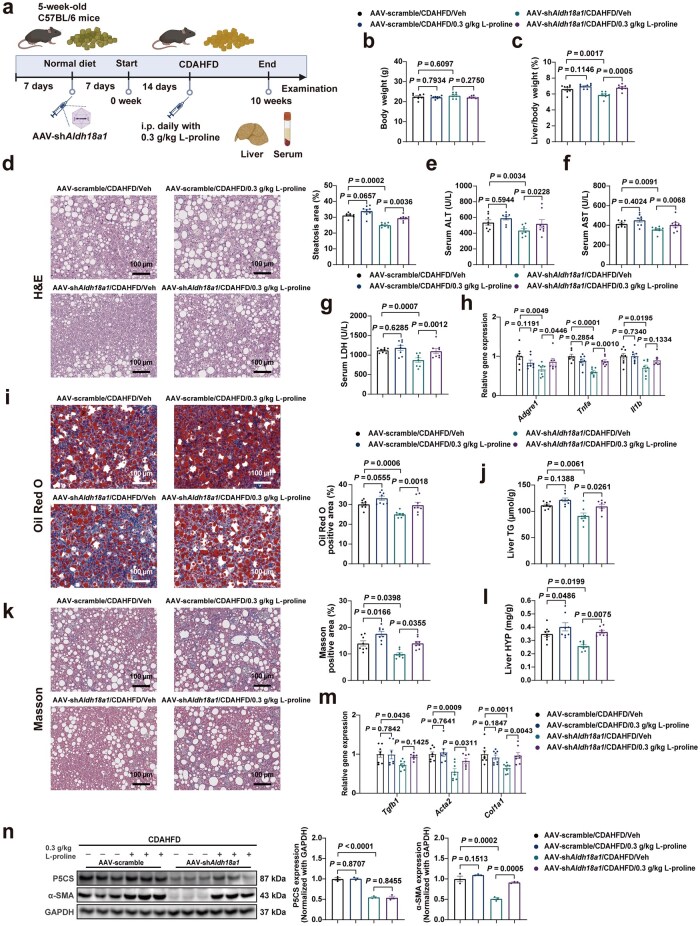
P5CS promotes the progression of MASLD via its downstream enzyme product proline. (a) Schematic diagram of experiment design to examine the function of proline replenishment in the liver-specific P5CS-knockdown CDAHFD-MASH mice. Mice injected with AAV-scramble and AAV-sh*Aldh18a1* were intraperitoneally administered by L-proline and fed with CDAHFD (*n *= 8 mice/group). (b) Body weight of mice in the indicated groups (*n *= 8 mice/group). (c) Liver/body weight ratio of mice in the indicated groups (*n* = 8 mice/group). (d) H&E staining (left) and steatosis area quantification (right) of liver sections from indicated groups of mice (*n *= 8 mice/group). Scale bar, 100 µm. (e–g) Serum ALT (e), AST (f), and LDH (g) levels of the indicated groups of mice (*n *= 8 mice/group). (h) Relative mRNA levels of hepatic proinflammatory genes in the indicated groups of mice (*n *= 8 mice/group). (i) Oil Red O staining (left) and quantification (right) of liver sections from indicated groups of mice (*n *= 8 mice/group). Scale bar, 100 µm. (j) Hepatic TG content of mice in the indicated groups (*n *= 8 mice/group). (k) Masson staining (left) and quantification (right) of liver sections from indicated groups of mice (*n *= 8 mice/group). Scale bar, 100 µm. (l) Hepatic HYP content of mice in the indicated groups (*n *= 8 mice/group). (m) Relative mRNA levels of hepatic fibrogenesis-related genes in the indicated groups of mice (*n *= 8 mice/group). (n) Immunoblot analysis (left) and quantification (right) of P5CS and α-SMA levels in the livers of mice in the indicated groups (*n *= 3 mice/group). Protein expression was normalized to GAPDH level. Data are shown as mean ± SEM. Two-way ANOVA was used for multi-group comparisons. The steatosis area, Oil Red O positive area, and Masson positive area were quantified by ImageJ software. ALT, ­alanine aminotransferase; AST, aspartate aminotransferase; CDAHFD, choline-deficient, L-amino acid-defined, high-fat diet; HYP, hydroxyproline; LDH, lactate dehydrogenase; TG, triglyceride.

To examine the function of proline replenishment in the liver-specific P5CS-knockdown CDAHFD-MASH mice, an animal experiment was designed as illustrated in Fig. 6a. No differences in final body weight were observed among each group of mice (Fig. 6b). Proline supplementation did not significantly increase lipid accumulation, liver injury, and inflammatory response in the livers of mice subjected to CDAHFD feeding conditions. However, proline supplementation inhibited the amelioration of hepatic steatosis, liver injury, and inflammatory response resulting from liver-specific P5CS knockdown. These were proved by the results of liver/body weight ratio (Fig. 6c), H&E staining (Fig. 6d), plasma concentrations of liver injury indicators (Fig. 6e–g), mRNA expression levels of proinflammatory genes (Fig. 6h), Oil Red O staining (Fig. 6i), and liver TG content (Fig. 6j). Moreover, cell damage resulting from PA-induced lipotoxicity in primary hepatocytes was reduced following P5CS knockdown. However, this effect was counteracted by proline replenishment (Supplementary Fig. S10a–d), which was consistent with the results of the previous *in vivo* experiments. The reduction of fibrosis in the livers of CDAHFD-induced MASH mice resulting from P5CS knockdown was also counteracted by proline supplementation, as evidenced by the results of Masson staining (Fig. 6k), liver HYP content (Fig. 6l), the expression levels of liver fibrogenesis-related genes (Fig. 6m), and α-SMA protein levels (Fig. 6n). Notably, the liver and serum proline contents reduced by P5CS knockdown were restored by proline replenishment (­Supplementary Fig. S10e and f), meaning that exogenous proline was effectively administered and might serve as a source for ­promoting MASLD progression. Consistently, the mRNA levels of genes related to liver fatty acid biosynthesis were unchanged across all groups (Supplementary Fig. S10g). However, proline supplementation reduced the elevated hepatic mRNA levels of genes related to fatty acid oxidation (Supplementary Fig. S10h) and mitochondrial function (Supplementary Fig. S10i) resulting from P5CS knockdown. In summary, these findings demonstrate that proline serves as the main downstream factor of P5CS in facilitating MASLD progression.

## Discussion

MASLD has evolved into a global pandemic, significantly impacting public health and imposing a substantial economic burden [4]. Although extensive basic and clinical research has been conducted on MASLD, current clinical treatment options remain limited [4, 9–11]. Our research determines that P5CS (*ALDH18A1*) overexpression is involved in the development of MASLD. P5CS deficiency ameliorates hepatic steatosis, inflammation, and fibrosis, indicating that P5CS may be the potential target for MASLD treatment.

Hepatic steatosis is regarded as the main cause for liver inflammation and fibrosis through lipotoxicity-induced hepatocyte apoptosis or necrosis [45]. Numerous preclinical and clinical-stage therapeutics specifically focus on target related to hepatic steatosis for intervention, including peroxisome proliferator-activated receptor (PPAR) [46], stearoyl-CoA desaturase-1 (SCD-1) [47], and patatin-like phospholipase domain-containing protein 3 (PNPLA3) [48]. Current evidence sufficiently demonstrates that improving hepatic lipid metabolic disorders can effectively halt MASLD progression. Previous research has reported that glutamine metabolism regulated by GLS1 is closely associated with MASLD via the regulation of mitochondrial lipid metabolism [34]. Moreover, it has been reported that P5CS is the rate-limiting enzyme in the proline biosynthetic pathway, as a gene that cells can downregulate in response to glutamine starvation in cancers [49], which builds a linkage among P5CS, glutamine, and hepatic lipid metabolism. As mentioned in our study, P5CS is dramatically upregulated in the development of MASLD. Furthermore, both *in vivo* and *in vitro* experiments exhibit that P5CS can regulate mitochondrial oxidation and lipid metabolism. Also, the inhibition of P5CS activity could enhance lipid oxidation and reduce hepatic lipid accumulation. Therefore, our results show that inhibition of P5CS could combat MASLD via the improvement of hepatic steatosis.

The urea cycle evolved to protect organisms against exposure to excess ammonia, a product of protein catabolism [50, 51]. Recent data show that patients with metabolic disorders, including obesity, MASLD, and T2DM, exhibit ureagenesis dysfunction. It has been demonstrated that impairment of urea cycle induced by hepatic arginase 2 deficiency causes hepatic steatosis via the induction of the impairment of TCA cycle, and mitochondrial dysfunction [52, 53]. P5CS is the key metabolic enzyme to regulate proline synthesis. In CDAHFD and HFHC-induced MASLD mouse models, the increase of hepatic proline level is observed. Once P5CS is knockdown in the liver, the hepatic proline level is decreased, and hepatic steatosis is improved. However, the protective effect of P5CS deficiency on MASLD mouse model is blocked by proline supplementation, suggesting that proline accumulation causes metabolic dysfunction. While P5CS is primarily responsible for proline biosynthesis, proline can also be derived from ornithine, a central amino acid in the urea cycle [54]. Combined with the effects of urea cycle dysfunction on MASLD, we suppose that P5CS-derived proline accumulation disturbs the urea cycle, leading to impairment of TCA cycle and mitochondrial dysfunction. However, the metabolic flux of urea cycle affected by P5CS remains to be determined.

The inhibition of P5CS has also been reported to suppress cancer cell proliferation of hepatocellular carcinoma (HCC), and the ­mechanism is demonstrated to be related to the enhancement of proline synthesis [55–57]. Aberrant lipid metabolism has been investigated to be closely associated with the progression of HCC, and modulating lipid metabolism could influence the progression of HCC through the regulation of metabolic enzymes, such as acetyl-CoA carboxylase (ACC) [58], ATP-citrate lyase (ACLY) [59], acyl-CoA ­synthetase long chain family member 3 (ACSL3) [60], and fatty acid synthase (FASN) [61]. In this article, the authors demonstrate that the P5CS–proline pathway promotes MASLD/MASH development by regulating hepatic lipid accumulation. Hence, the authors provide a new insight into the effects of P5CS-coupled proline metabolism, as modulating proline metabolism and lipid metabolism have been demonstrated to have the potential to influence HCC progression. While the high expression level of P5CS (*ALDH18A1*) and dysregulated proline metabolism have been observed in HCC, the underlying mechanism of its role in MASLD-to-HCC progression is unclarified. Given that MASLD is a primary risk factor for HCC [6], the link between P5CS, lipid metabolism, and proline metabolism indicates that the proline metabolism disorder related to abnormal P5CS expression might not only contribute to MASLD but also influence the development of MASLD to HCC. ­Furthermore, the effects of P5CS on lipid metabolism might be the potential mechanism involved in MASLD-to-HCC progression.

In summary, this study establishes a close linkage between P5CS and hepatic lipid metabolism. Across various animal models and patients, the authors identify specific overexpression of P5CS and demonstrate that its deficiency exerts beneficial effects in ameliorating MASLD. Furthermore, the enzymatic mutants of P5CS were shown to recapitulate the lipid metabolism-improving effects observed in P5CS-deficient models, indicating that targeting P5CS activity ­represents a viable therapeutic strategy for MASLD intervention.

### Limitations of the study

This study demonstrates that P5CS promotes MASLD progression in male mice, but whether its function could be observed in female mice and in nonhuman primates remains unclear. Moreover, the authors reveal that the addition of L-proline could block the ­beneficial mitochondrial-protective effects resulting from the P5CS knockdown; however, how proline itself affects mitochondrial functions needs more investigation. Finally, the *in vivo* function of the mutant P5CS under disease conditions needs to be explored and validated.

## Materials and methods

### Animal studies

All animal experiments and protocols employed in this work received approval from the Animal Ethics Committee of the Shanghai ­Institute of Materia Medica, Chinese Academy of Sciences. All C57BL/6J male mice (5 weeks old, 18–22 g) were housed in isolated ventilated cages within an animal barrier facility at the Shanghai Institute of Materia Medica, Chinese Academy of Sciences (­Shanghai, China). The mice were maintained on a 12 h light/12 h dark cycle at 22–26 °C with access to sterile pellet food and water *ad ­libitum*. Mice were randomly allocated to treatment groups, thereafter, verifying identical body weight before treatment. A MASH mouse model was established by feeding male mice aged 6–7 weeks a CDAHFD (60 kcal% fat, 0.1% methionine, and no added choline; #A06071302, Research Diets, New Brunswick, USA) diet or an HFHC (40 kcal% fat, 20–22 kcal% fructose, and 2% cholesterol; #D09100310, Research Diets, New Brunswick, USA) diet. The control group consisted of mice that were given a normal diet (#Q031, Shanghai Shilin Biologic Science & Technology, Shanghai, China). For liver-specific AAV8 transduction, AAV-Vector/AAV-*ALDH18A1* or AAV-scramble/AAV-sh*Aldh18a1* (1.2 × 10^11^ genome copies/mouse; Genomeditech, Shanghai, China) was delivered by tail-vein injection. Then mice were fed with a normal diet or CDAHFD diet before being sacrificed for analysis. To observe the effects of proline on normal mice, treatment with L-proline (#HY-Y0252, MCE) at a dose of 0.1 g/kg/day and 0.3 g/kg/day were performed in C57BL/6J mice by intraperitoneal injection. To examine the function of proline replenishment in liver-specific P5CS-knockdown CDAHFD-MASH mice, following AAV-scramble/AAV-sh*Aldh18a1* transduction, mice were fed with CDAHFD for 2 weeks. They were subsequently given intraperitoneal L-proline at a dose of 0.3 g/kg/day until being ­sacrificed for analysis.

### Human studies

Human plasma and liver samples were collected from Shanghai Yangpu District Central Hospital during our research. All reported investigations were conducted in accordance with the principles of the Declaration of Helsinki and Istanbul and received approval from the Hospital’s Ethical Committee responsible for research (Approval no. LL-2023-SCI-019). Informed permission in writing was acquired from all subjects.

### Mouse liver function and serum assays

To evaluate liver injury, plasma levels of ALT, AST, and LDH in mice were measured using an Olympus AU 600 auto-analyzer (Olympus, Japan) in accordance with the manufacturer’s instructions.

### Measurement of hepatic TG content

Liver tissues (20–30 mg) were homogenized with 0.4 mL phosphate-buffered saline (PBS) in order to quantify intrahepatic TG content. Total liver TG was then extracted using 1.4 mL chloroform:ethanol (2:1 v/v) overnight. After centrifugation, the liquid phase at the bottom was transferred to a sterile tube and evaporated for 6–8 h. Subsequently, 1 mL of ethanol containing 1% Triton X-100 was used to dissolve TG. Using a TG Content Determination Kit (#1.02.1803; Fosun Pharmaceutical, China), the concentration of TG was measured and normalized to tissue weight.

### Measurement of hepatic HYP content

For liver HYP content measurement, 100 mg liver tissues were homogenized in 5 mL of 50% HCl solution and subsequently incubated at 120 °C for 24 h. The samples underwent filtering using a 0.45-μm microfiltration membrane, and 100 μL of the samples were collected and dried at 40°C for a duration of 72 h. After that, each sample was mixed with 1.2 mL of 50% isopropanol and 200 μL of a chloramine T working solution (1.8% trisodium citrate dihydrate, 0.3% citric acid, 2.8% sodium acetate, 19.2% isopropanol, and 0.6% chloramine T) at room temperature for 10 min. Then, 1 mL of the Ehrlich working solution (13.6% dimethylaminobenzaldehyde, 14.9% perchloric acid, and 73% isopropanol) was added to each sample followed by a 1.5-h reaction in a 50 °C water bath. The concentration of HYP was determined at 558 nm using a spectrophotometer (SpectraMax M5, Molecular Devices, Santa Clara, CA, USA) and normalized to tissue weight.

### Histological analysis

Mouse liver tissues were collected from the left lateral lobe and fixed in 4% paraformaldehyde overnight to perform histological analysis. To evaluate the patterns of steatosis and the extent of inflammation, liver sections were fixed, paraffin-embedded, and subsequently stained with H&E. To evaluate the pattern of liver fibrosis, liver sections were first fixed in paraffin and subsequently stained with Masson. Oil Red O staining of frozen liver sections produced in Tissue-Tek OCT compound was used to visualize lipid droplet accumulation. The ImageJ software was used for all quantitative analysis.

### Immunohistochemistry

For IHC, sections of human and mouse liver embedded in paraffin were immunostained. The sections were processed for antigen retrieval in a microwave and then blocked with 5% BSA (#A8020, Solarbio, Beijing, China) for 30 min at room temperature after quenching the endogenous peroxidase activity by incubating them in 3% H_2_O_2_ (#10011218, Sinopharm, Beijing, China) for 30 min at 37 °C. Anti-P5CS primary antibody (1:200, #17719-1-AP, Proteintech) was used to detect the expression of indicated protein. The sections were visualized using 3,3’-diaminobenzidine (DAB, #K5007, DAKO) after incubation with secondary antibodies conjugated with horseradish peroxidase. Finally, hematoxylin staining, dehydration, and mounting for bright-field microscopy were performed on the slides.

### Isolation and culture of primary hepatocytes

Mouse primary hepatocytes were isolated from mice aged 6–10 weeks and cultured. Briefly, mice were anesthetized and perfused through the portal vein with a perfusion medium, followed by perfusion with a liver-digesting medium containing collagenase type I (#LS004196, Worthington, Lakewood). After digestion, the liver was filtered through a 70-µm cell strainer (#15-1070, Biologix), subsequently collected, and added into the percoll purification solution (#P1644, Sigma-Aldrich). After centrifugation at 700 *g* for 10 min and removal of other types of cells and cell debris in the supernatant, the obtained primary hepatocytes were resuspended in HepatoZYME-SFM (#17705021, GIBCO, Grand Island, NY, USA) supplemented with 10% fetal bovine serum (#10091-148, Gibco) and 1% penicillin/streptomycin before being seeded in ­culture plates. To establish an FFA-induced model of lipid accumulation in hepatocytes, a mixture of PA (0.1 mmol/L, #57-10-3, Sigma-Aldrich) and oleic acid (OA; 0.2 mmol/L, #112-80-1, Sigma-Aldrich) in 0.5% BSA was added to the culture medium for 24 h. To induce cell damage of hepatocytes, hepatocytes were treated with 0.4 mmol/L PA or 0.5% BSA vehicle for 24 h before collecting the cell supernatant.

### AML12 cell culture

AML12 cells were purchased from the American Type Culture ­Collection (ATCC, # CRL-2254; Research Resource Identifier: CVCL_0140) and cultured in accordance with American Type Culture Collection guidelines. To induce lipid accumulation in AML12 cells, culture media was supplemented with 0.3 mmol/L FFA (0.1 mmol/L PA and 0.2 mmol/L OA mixed in 0.5% BSA), followed by a 24-h incubation. In order to analyze signal transduction and measure mitochondrial respiration rates of AML12 cells, cells were treated with 0.4 mmol/L PA or 0.5% BSA vehicle before analysis.

### Cell transfection with plasmids or small interfering RNA

The plasmids used in this work were obtained from Youbio ­Biotechnology (Changsha, China). The primers used for plasmid construction are given in Supplementary Table S1. AML12 cells or primary hepatocytes were transfected with the desired plasmids using Lipofectamine 3000 transfection reagent (#L3000015, Thermo Fisher Scientific) in Opti-MEM (#31985070, Invitrogen, USA), following the manufacturer’s instructions. After 6–8 h of incubation, the medium was replaced with complete medium, and the cells were subsequently incubated for 24 h before further treatment. For small interfering RNA (siRNA) transfection, AML12 cells or primary hepatocytes were transfected with siRNA (GenePharma, Shanghai) specifically targeting *Aldh18a1* using Lipofectamine RNAi MAX (#13778075, Thermo Fisher Scientific) in accordance with the manufacturer’s guidelines. The antisense siRNA sequences targeting *Aldh18a1* are given in Supplementary Table S1. Following a 24-h transfection period in complete medium, the cells were subjected to the aforementioned treatment.

### Western blot analysis

Cells were lysed, sonicated, and subjected to boiling at 100 °C for 10 min in a sample buffer consisting of 50 mmol/L Tris-HCl, 2% w/v SDS, 10% glycerol, 1% β-mercaptoethanol, and 0.01% bromophenyl blue (pH 6.8). After that, cell lysates were separated on sodium dodecyl sulfate–polyacrylamide gel electrophoresis (SDS–PAGE) and subsequently transferred to nitrocellulose filter membranes. The membranes were then incubated with blocking buffer (TBS with 0.01% Tween 20 and 5% non-fat milk) for 1 h at room temperature, followed by an overnight incubation at 4 °C in a buffer containing primary antibodies (the primary antibodies used are given in ­Supplementary Table S2). The membranes were washed three times and subsequently incubated with secondary antibodies for 1 h at room temperature. Following three washes, immunostaining was visualized using Azure and ChemiDoc imaging system (Bio-Rad). Tissue samples were lysed using RIPA Lysis Buffer (#P0013B, Beyotime Biotechnology) on ice for 30 min, followed by centrifugation. The supernatants were boiled in sample buffer and analyzed as previously described.

### RNA isolation and RT-qPCR

Total RNA was extracted by homogenizing snap-frozen tissues or cultured hepatocytes in TRIzol reagent (#15596018, Life Technologies) following the manufacturer’s instructions. cDNA was synthesized using the ABScript III RT Master Mix (#RK20428, ABclonal). qPCR was performed with a QuantStudio system (Thermo Fisher Scientific) using 2× Universal SYBR Green Fast qPCR Mix (#RK21203, ABclonal) and specific primer pairs. The data were then normalized to the expression of housekeeping gene (*GAPDH* or 18S rRNA). The list of primers used is shown in Supplementary Table S2.

### Measurement of cellular TG content

Cells were lysed using RIPA Lysis Buffer (#P0013B, Beyotime ­Biotechnology) on ice for 30 min, followed by ultrasonic disruption and centrifugation. TG concentration in the supernatants was measured using the TG Content Determination Kit (#1.02.1803, Fosun Pharmaceutical, China) following the manufacturer’s instructions and subsequently normalized to the total cellular protein content.

### Proline assay

Proline content was determined with a validated proline assay kit (#BC0295, Solarbio), with all samples extracted in accordance with the manufacturer’s instructions. Briefly, cells were lysed using ­sonication, whereas liver tissues were lysed using a grinder. Then supernatants were shaken and extracted in a boiling water bath for 10 min. Following centrifugation at 10,000 *g* for 10 min at room temperature, the supernatants were collected for measurement. Absorbance at 520 nm was used to determine the concentration of proline, and all results were normalized to the total cellular protein content or tissue weight.

### Mitochondrial respiration analysis

Mitochondrial respiratory functions were assessed by analyzing oxygen consumption rates (OCRs) with the Seahorse XFe24 Analyzer (Agilent Technologies, CA, USA). Briefly, AML12 cells were transfected with plasmids or siRNA for 24 h, thereafter trypsinized, collected, and plated to near confluency at 30,000 cells per reaction well in a 24-well XF Seahorse cell culture microplate (#100777-004, Agilent Technologies, CA, USA). Before measurements on the day of the experiment, AML12 cells were treated with either 0.4 mmol/L PA or 0.5% BSA vehicle for 16 h. During the experiment, the cells were equilibrated in XF Base Medium (#103575-100, Agilent) supplemented with 10 mmol/L glucose, 2 mmol/L sodium pyruvate, and 2 mmol/L L-glutamine (#103577-100, #103578-100, and #103579-100, Agilent). The culture microplate was subsequently placed in a non-CO_2_ incubator for 1 h at 37°C. The injection protocol consisted of three steps, with at least three replicate measurements taken between each compound injection. After the recording of the baseline OCR, continuous injections of pharmacologic inhibitors were administered through ports in the XF Assay cartridges: oligomycin (1 µmol/L), an ATP synthase inhibitor that enables the ­measurement of ATP-coupled oxygen consumption via oxidative phosphorylation (OXPHOS); carbonyl cyanide 4-trifluoromethoxy-phenylhydrazone (FCCP, 1 µmol/L), an uncoupling agent that ­generates maximum electron transport, thereby allowing the ­measurement of maximum OXPHOS respiration capacity; and a mixture of antimycin A (1 µmol/L) and rotenone (1 µmol/L), which inhibit mitochondrial complex I and III, respectively. The raw data from all experiments were analyzed using Agilent Wave Desktop Software version 2.6.3.5 (Agilent). Finally, the measurement results of cellular oxygen consumption were normalized to the total ­protein content of each well.

### Stable isotope-assisted tracing

si*Aldh18a1* and negative control siRNA were transfected into AML12 cells for 24 h. Then, the cells were treated with 0.4 mmol/L PA for 4 h and then incubated in serum-free medium containing 0.4 mmol/L [U–^13^C_16_]-PA (#CLM-6059-1, Cambridge Isotope ­Laboratories, Inc.) for 12 h. Subsequently, the cells were trypsinized, centrifuged, collected, and preserved in liquid nitrogen. The cellular contents of M + 2 TCA cycle metabolites were obtained from metabolic flux analysis (Shanghai Institute of Materia Medica-Servier Joint Laboratory, China). Finally, the original data were corrected for natural isotopes and analyzed.

### RNA-seq and data analysis

Liver samples from mice fed a normal diet (*n *= 3) or CDAHFD (*n *= 3) were sent to Majorbio (Shanghai, China) for RNA-seq transcriptome sequencing and data analysis utilizing the Illumina NovaSeq6000 platform. All sequencing data are accessible in GEO datasets (GEO: GSE295909). Differentially expressed genes between the two groups were identified by the DEseq2 algorithm. Genes with a false-adjusted *P* value < 0.05 and a log_2_-fold change value ≥ 1 or ≤ −1 were considered as significantly changed.

### Statistics

All data are presented as the mean ± SEM and were statistically analyzed with GraphPad Prism version 9.0 (GraphPad Software Inc, USA). An unpaired, two-tailed Student’s *t*-test was used for comparisons between two groups, while one-way or two-way analysis of variance was used for comparisons among multiple groups, followed by a *post-hoc* least significant difference (LSD) test to determine the differences between any two groups. A *P* value of less than 0.05 was considered as statistically significant.

## Supplementary Material

loaf040_Supplementary_Data

## Data Availability

All the data supporting the findings of this study are available within the Supplementary material and corresponding authors.
