# Supplementary material for: P5CS-coupled proline metabolism manipulates metabolic dysfunction-associated steatotic liver disease
Source: Life Metab. 2025 Nov 19;5(2):loaf040. doi: 10.1093/lifemeta/loaf040 (PMC13110119; doi:10.1093/lifemeta/loaf040)
Supplement: loaf040_Supplementary_Data [file loaf040_supplementary_data.zip › 05-Dec-2025_043702_Supplementary_material.docx]

Supplementary Information for

**P5CS coupled proline metabolism manipulates metabolic dysfunction-associated steatotic liver disease**

Zan Lyu^1,2^, Sike Yu^3^, Chang Peng^3^, Wenbiao Wu^3^, Wenhua Yang^4^, Huan Ma^2^, Yan Sun^4^, Liya Jing^4^, Hongyu Gu^4^, Erjiang Tang^5,6^, Xuemei Zhang^1,7,^
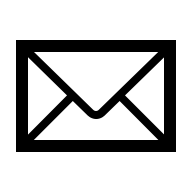
, Huihong Jiang^5,6,^
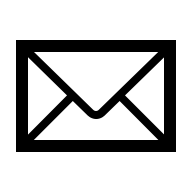
, Haowen Jiang^4,^
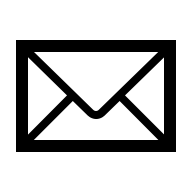
, Jia Li^2,3,4,8,9,^
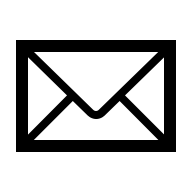


1. Department of Pharmacology, School of Pharmacy, Fudan University, Shanghai 201203, China
2. State Key Laboratory of Chemical Biology, Shanghai Institute of Materia Medica, Chinese Academy of Sciences, Shanghai 201203, China
3. Key Laboratory of Glyco-drug Research of Zhejiang Province, School of Pharmaceutical Science and Technology, Hangzhou Institute for Advanced Study, University of Chinese Academy of Sciences, 310024, Hangzhou, China
4. School of Life Science and Technology, Shanghai Tech University, Shanghai 201210, China
5. Department of General Surgery, Yangpu Hospital, School of Medicine, Tongji University, Shanghai, 200090, China
6. Center for Clinical Research and Translational Medicine, Yangpu Hospital, School of Medicine, Tongji University, Shanghai, 200090, China
7. School of Pharmacy, East China Normal University, Shanghai 200241, China
8. Zhongshan Institute for Drug Discovery, Shanghai Institute of Materia Medica, Chinese Academy of Sciences, Zhongshan Tsuihang New District, Guangdong 528400, China
9. Shandong Laboratory of Yantai Drug Discovery, Bohai Rim Advanced Research Institute for Drug Discovery, Yantai, Shandong 264117, China


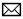
Correspondence: Xuemei Zhang: [xuemzhang@fudan.edu.cn](mailto:xuemzhang@fudan.edu.cn); Huihong Jiang: jiang572579@163.com; Haowen Jiang: haowenjiang@simm.ac.cn; Jia Li: [jli@simm.ac.cn](mailto:jli@simm.ac.cn)

**This file includes:**

Supplementary Figures S1-S10

Supplementary Tables S1-S2

**Supplementary Figures S1-S10**


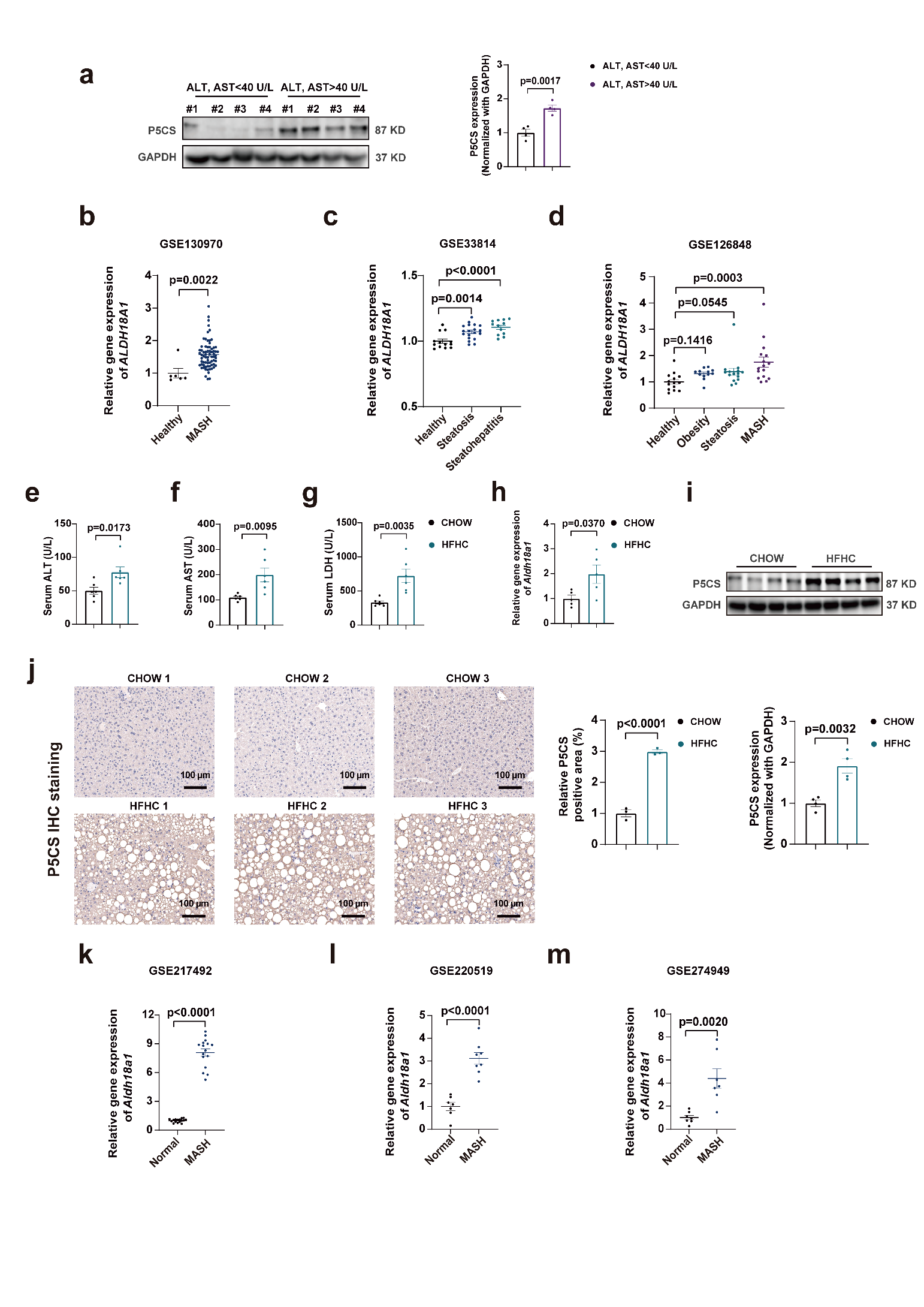


**Supplementary Figure S1. P5CS (*ALDH18A1*) expression is upregulated during the progression of MASLD.**

(a) Immunoblot analysis (left) and quantification (right) of P5CS expression in the livers of clinical patients (n = 4 patients/group). Protein expression was normalized to GAPDH level. (b-d) Analysis of the relative hepatic *ALDH18A1* mRNA expression level in GSE130970 (b), GSE33814 (c), and GSE126848 (d) public datasets. (e-g) Serum ALT (e), AST (f), and LDH (g) levels of CHOW- or HFHC-fed mice (n = 6 mice/group). (h) Relative mRNA level of *Aldh18a1* in the livers of CHOW- or HFHC-fed mice (n = 5 mice/group). (i) Immunoblot analysis (upper) and quantification (bottom) of P5CS expression in the livers of CHOW- or HFHC-fed mice (n = 3 mice/group). Protein expression was normalized to GAPDH level. (j) IHC staining (left) and quantification (right) of hepatic P5CS expression in the livers of CHOW- or HFHC-fed mice (n = 3 mice/group) Scale bar, 100 µm. (k-m) Analysis of the relative *Aldh18a1* mRNA expression level in the livers of diet-induced MASH mice in GSE217492 (k), GSE220519 (l), and GSE274949 (m) public datasets. Data are shown as mean ± SEM. Two-tailed Student’s t test was used for two-group comparisons, and one-way ANOVA was used for multi-group comparisons. IHC positive area was quantified by Image J software. ALT, alanine aminotransferase; AST, aspartate aminotransferase; HFHC, high-fat/high-cholesterol, high carbohydrate; LDH, lactate dehydrogenase.


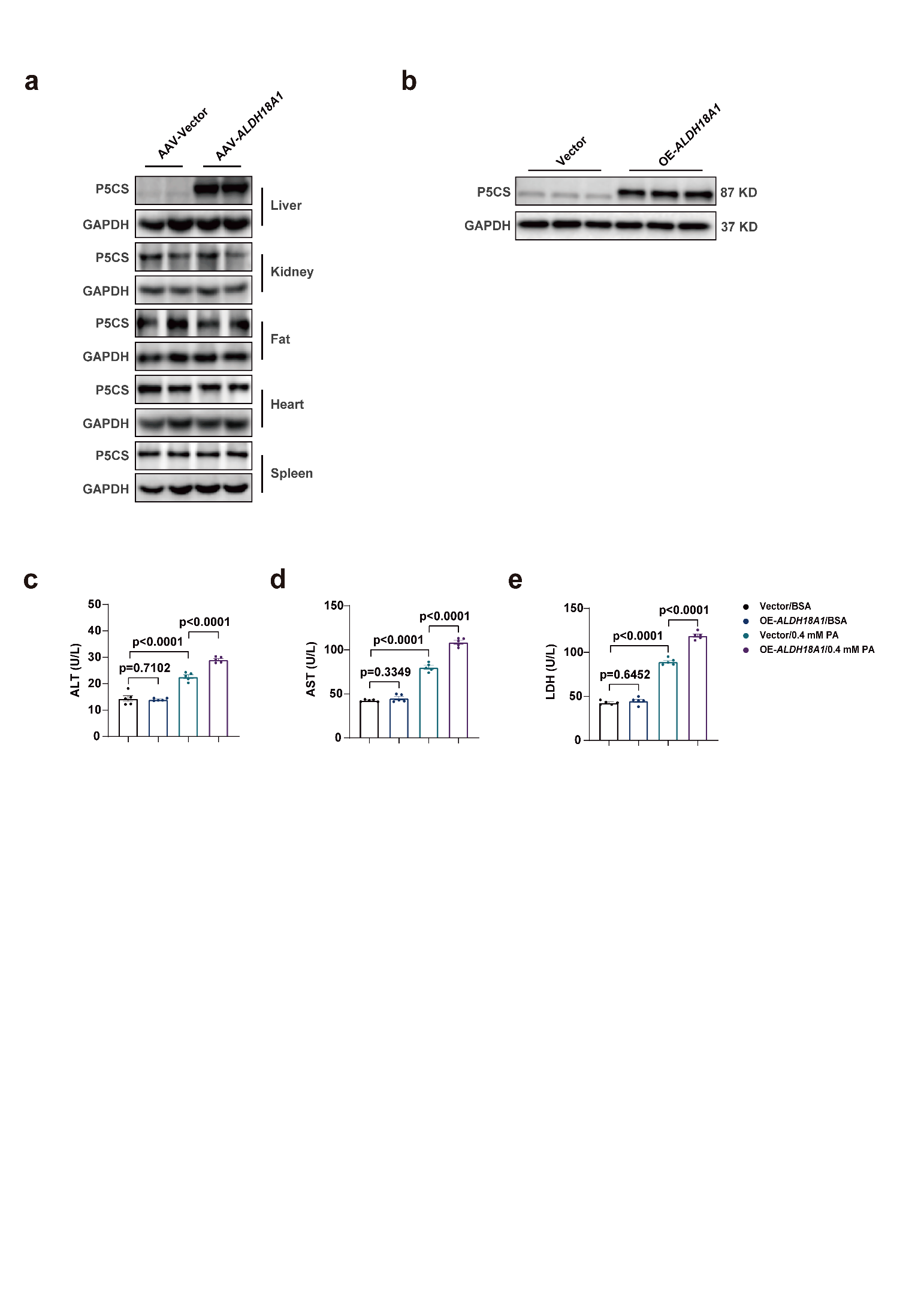


**Supplementary Figure S2. *In vivo* efficiency of the P5CS overexpression vector, and the impact of P5CS overexpression on PA-induced cellular lipotoxicity.**

(a) The P5CS expression levels in liver and other tissues after liver-specific P5CS overexpression in mice. (b) The P5CS expression level in primary hepatocytes transfected with Vector or *ALDH18A1* plasmid. (c-e) ALT (c), AST (d), and LDH (e) concentrations in the cell supernatants of primary hepatocytes transfected with Vector or *ALDH18A1* plasmid followed by treatment with BSA or PA (n = 5). Data are shown as mean ± SEM. Two-way ANOVA was used for multi-group comparisons. ALT, alanine aminotransferase; AST, aspartate aminotransferase; LDH, lactate dehydrogenase; PA, palmitic acid.


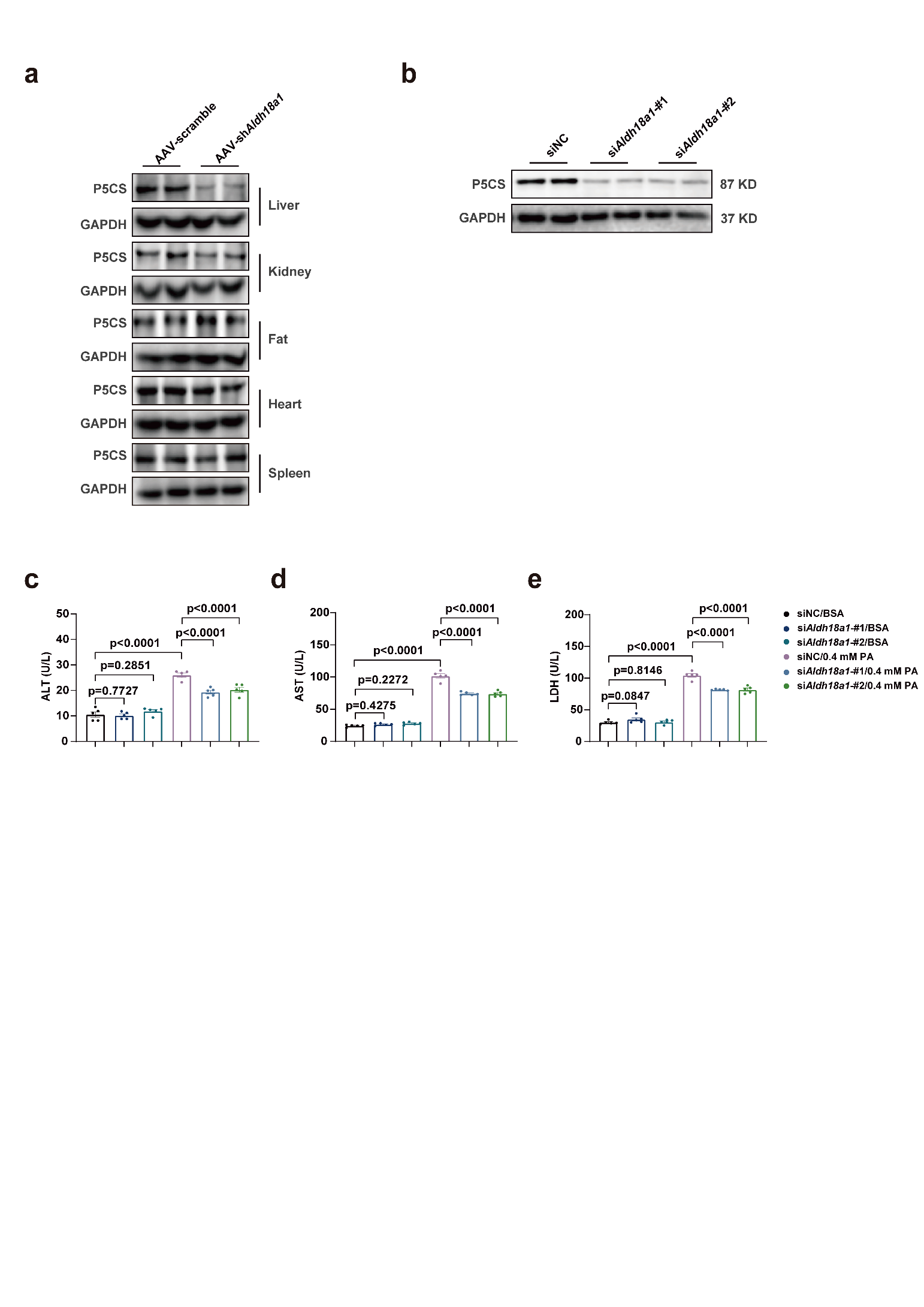


**Supplementary Figure S3. *In vivo* efficiency of the *Aldh18a1* shRNA, and the impact of P5CS knockdown on PA-induced cellular lipotoxicity.**

(a) The P5CS expression levels in liver and other tissues after liver-specific P5CS knockdown in mice. (b) The P5CS expression level in primary hepatocytes transfected with negative control siRNA or si*Aldh18a1*. (c-e) ALT (c), AST (d), and LDH (e) concentrations in the cell supernatants of primary hepatocytes transfected with negative control siRNA or si*Aldh18a1* followed by treatment with BSA or PA (n = 5). Data are shown as mean ± SEM. Two-way ANOVA was used for multi-group comparisons. ALT, alanine aminotransferase; AST, aspartate aminotransferase; LDH, lactate dehydrogenase; PA, palmitic acid.


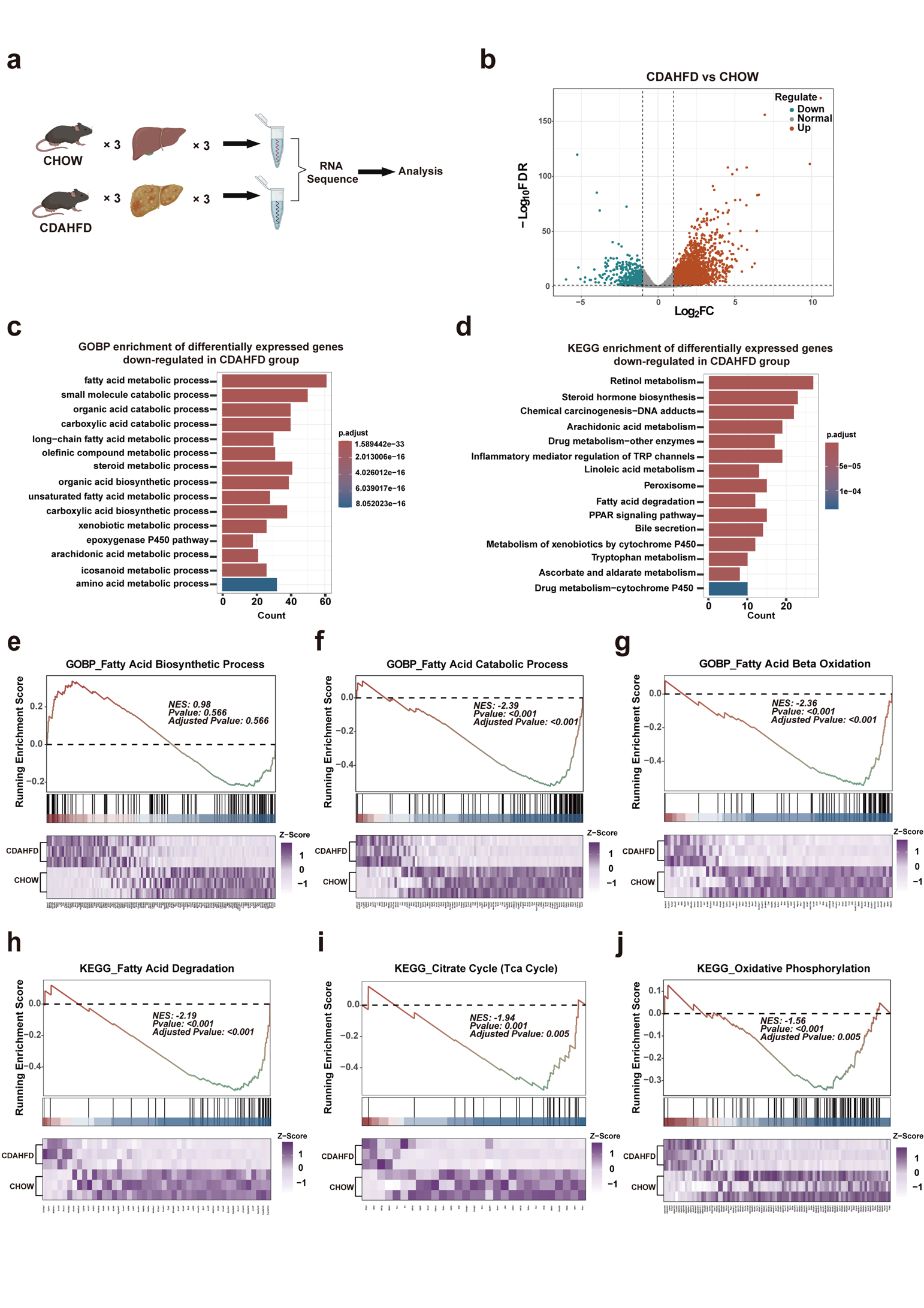


**Supplementary Figure S4. Fatty acid catabolism and mitochondrial function pathways are downregulated in the livers of mice fed with CDAHFD.**

(a) Schematic diagram of performing RNA-seq for the livers of CHOW- or CDAHFD-fed mice. (b) Volcano plot of the differentially expressed genes in mice livers with CDAHFD consumption. (c-d) GO-BP (c) and KEGG (d) enrichment analysis of differentially expressed genes downregulated in the livers of mice fed with CDAHFD. (e-j) GSEA analysis of the changes in liver fatty acid biosynthesis (e), fatty acid oxidation (f-h), and mitochondrial function (i and j) pathways. CDAHFD, choline-deficient, L-amino acid-defined, high-fat diet.


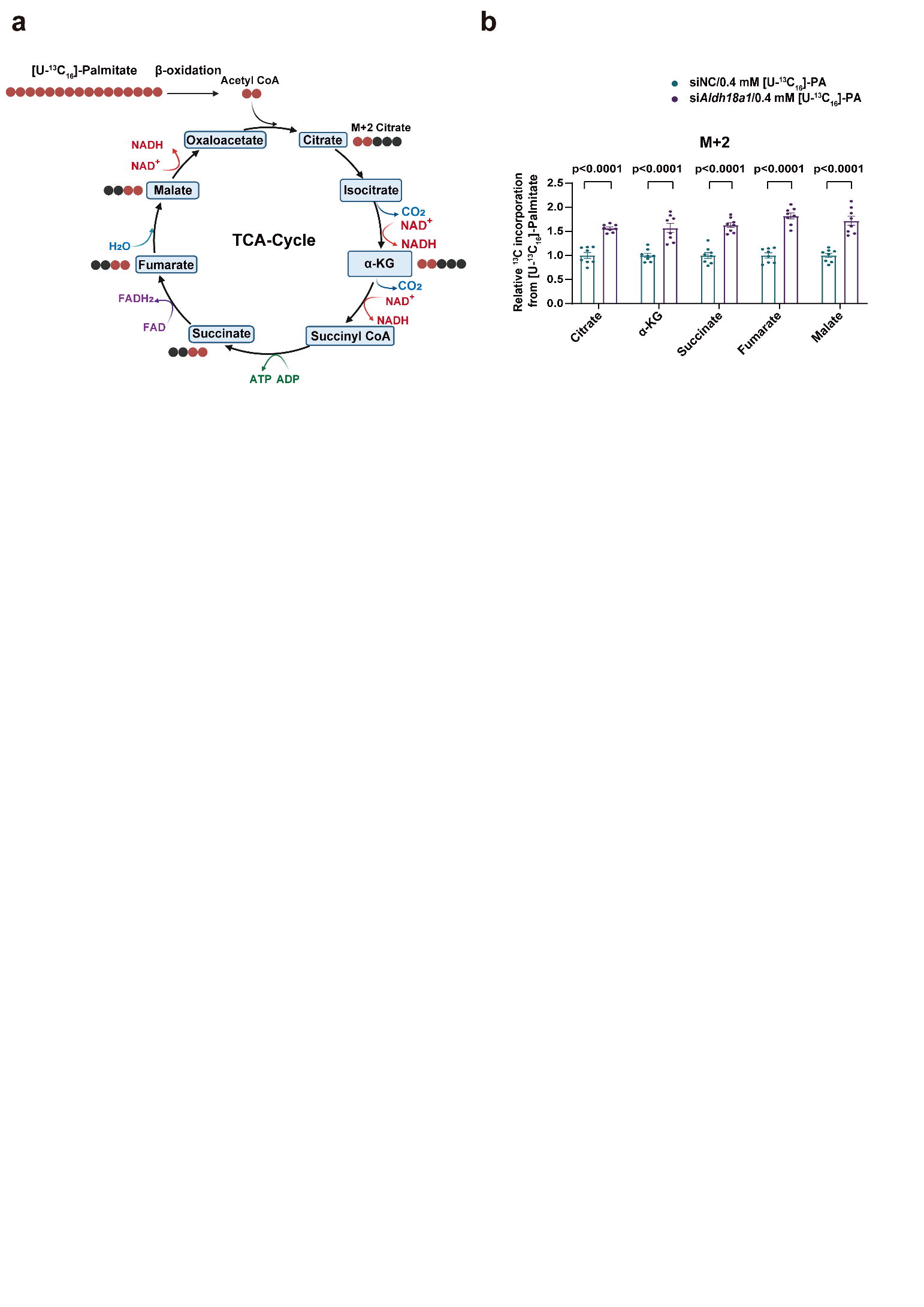


**Figure S5. Influence of P5CS on the hepatocyte mitochondrial TCA cycle.**

(a) Schematic diagram of [U-^13^C_16_]-PA labeling and tracing within AML12 cells. (b) Relative abundance of TCA cycle-related M+2 metabolites derived from [U-^13^C_16_]-PA in AML12 cells transfected with negative control siRNA or si*Aldh18a1* (n = 8). Data are shown as mean ± SEM. Two-tailed Student’s t test was used for two-group comparisons. α-KG, α-Ketoglutaric acid; PA, palmitic acid.


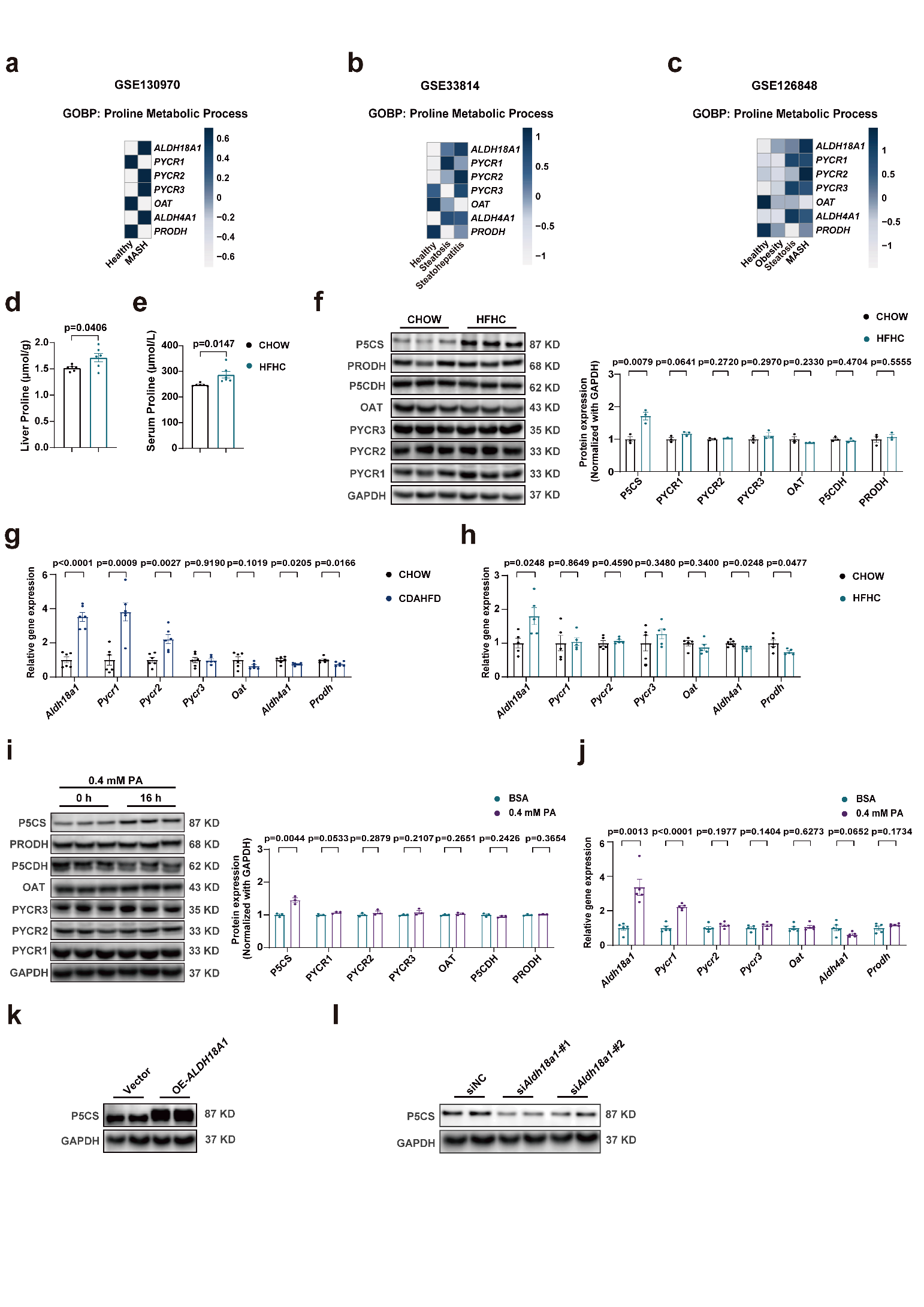


**Supplementary Figure S6.** **P5CS coupled proline metabolism is disrupted during MASLD progression.**

(a-c) Heatmap analysis of the proline metabolism-related genes in GSE130970 (a), GSE33814 (b), and GSE126848 (c) public datasets. (d) The liver proline content of CHOW- or HFHC-fed mice (n = 6 mice/group). (e) The serum proline content of CHOW- or HFHC-fed mice (n = 6 mice/group). (f) Immunoblot analysis (left) and quantification (right) of the expressions of proline metabolism-related proteins in the livers of CHOW- or HFHC-fed mice (n = 3 mice/group). Protein expression was normalized to GAPDH level. (g) Relative mRNA levels of genes related to proline metabolism in the livers of CHOW- or CDAHFD-fed mice (n = 6 mice/group). (h) Relative mRNA levels of genes related to proline metabolism in the livers of CHOW- or HFHC-fed mice (n = 5 mice/group). (i) Immunoblot analysis (left) and quantification (right) of the expressions of proline metabolism related-proteins in BSA- or PA-treated AML12 cells (n = 3). Protein expression was normalized to GAPDH level. (j) Relative mRNA levels of genes related to proline metabolism in BSA- or PA-treated AML12 cells (n = 5). (k) The P5CS expression level in AML12 cells transfected with Vector or *ALDH18A1* plasmid. (l) The P5CS expression level in AML12 cells transfected with negative control siRNA or si*Aldh18a1*. Data are shown as mean ± SEM. Two-tailed Student’s t test was used for two-group comparisons. CDAHFD, choline-deficient, L-amino acid-defined, high-fat diet; HFHC, high-fat/high-cholesterol, high carbohydrate; PA, palmitic acid.


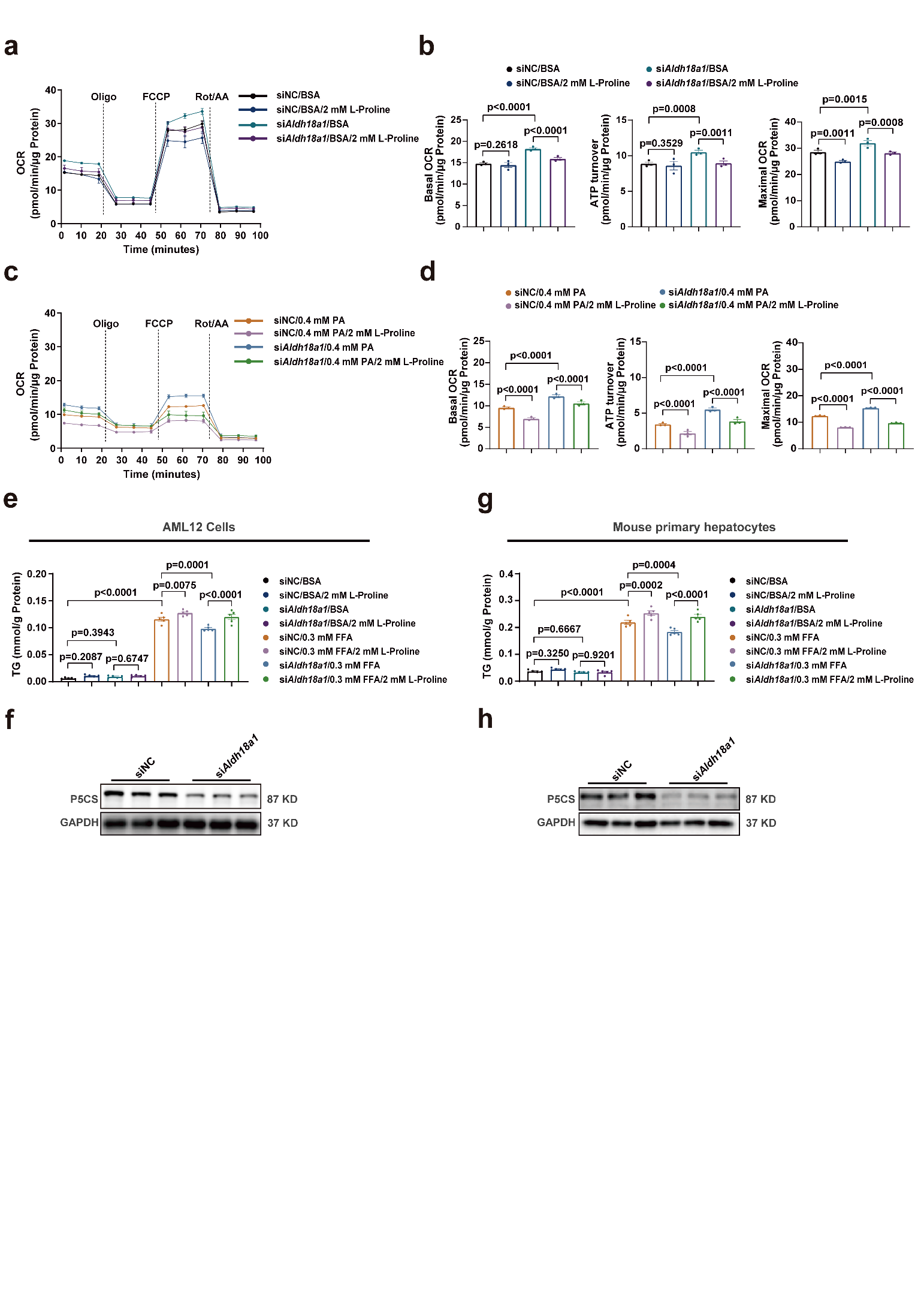


**Supplementary Figure S7. P5CS impairs hepatocyte mitochondrial function and exacerbates lipid accumulation through its downstream enzyme product proline.**

(a) Cellular respiration of Negative-control and P5CS-knockdown AML12 cells exposed to BSA followed by treatment with or without L-proline. (b) Basal, ATP-linked, and maximal OCRs of (a) measured by seahorse analysis (n = 4). (c) Cellular respiration of Negative-control and P5CS-knockdown AML12 cells exposed to PA followed by treatment with or without L-proline. (d) Basal, ATP-linked, and maximal OCRs of (c) measured by seahorse analysis (n = 3-4). (e) Cellular TG content of Negative-control and P5CS-knockdown AML12 cells exposed to BSA or FFA followed by treatment with or without L-proline (n = 5). (f) The P5CS expression level in AML12 cells transfected with negative control siRNA or si*Aldh18a1*. (g) Cellular TG content of Negative-control and P5CS-knockdown primary hepatocytes exposed to BSA or FFA followed by treatment with or without L-proline (n = 5). (h) The P5CS expression level in primary hepatocytes transfected with negative control siRNA or si*Aldh18a1*. Data are shown as mean ± SEM. Two-way ANOVA was used for multi-group comparisons. FFA, free fatty acid; OCR, oxygen consumption rate; PA, palmitic acid; TG, triglyceride.


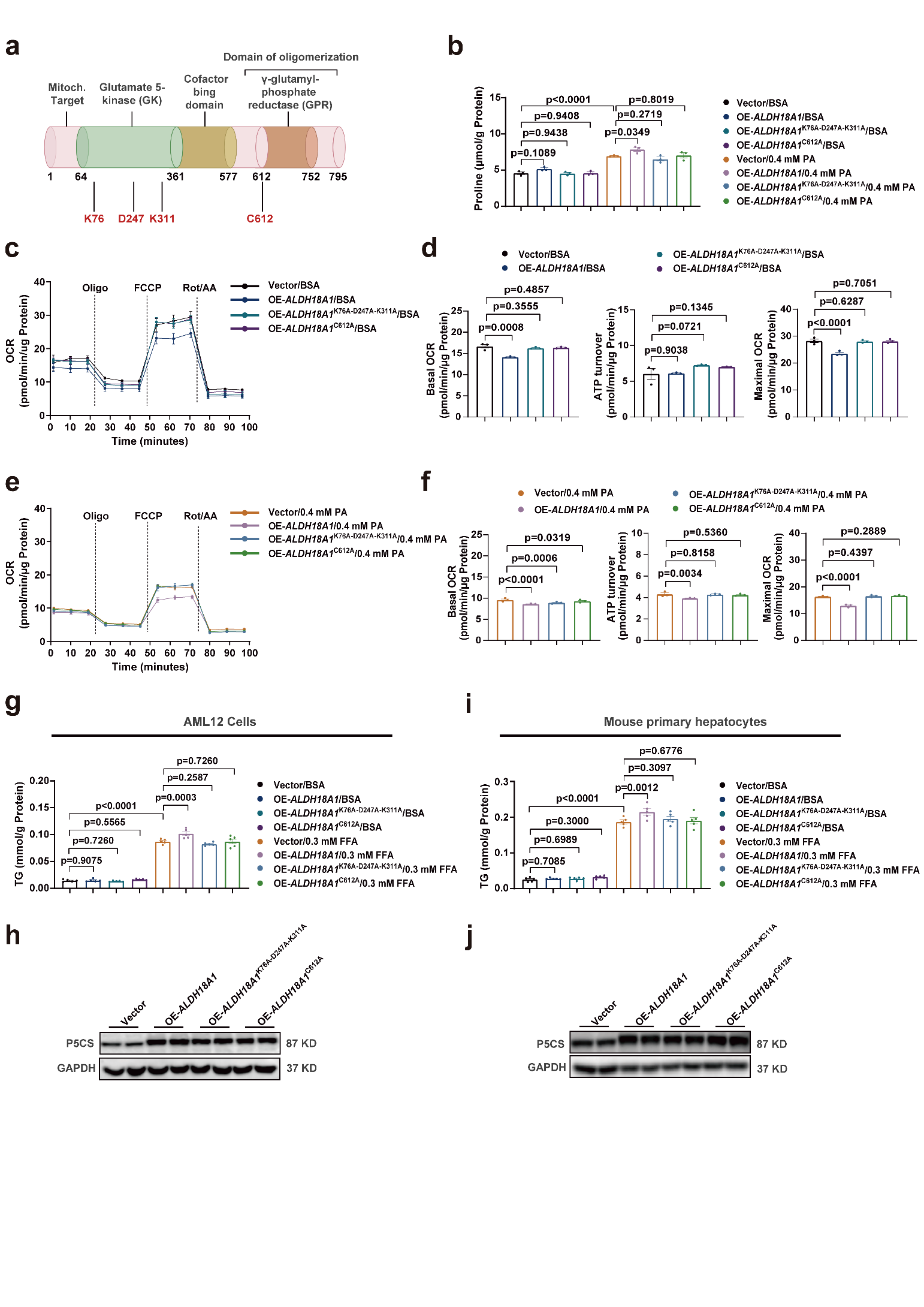


**Supplementary Figure S8.** **Enzyme activity is required for the cellular function of P5CS.**

(a) The schematic diagram indicated the domain organization of P5CS, and the residues mutated in this study. (b) The intracellular proline content of Vector-, WT-P5CS-, and mutant-P5CS-overexpressed AML12 cells treated with BSA or PA (n = 3). (c) Cellular respiration of Vector-, WT-P5CS-, and mutant-P5CS-overexpressed AML12 cells treated with BSA. (d) Basal, ATP-linked, and maximal OCRs of (c) measured by seahorse analysis (n = 3-4). (e) Cellular respiration of Vector-, WT-P5CS-, and mutant-P5CS-overexpressed AML12 cells treated with PA. (f) Basal, ATP-linked, and maximal OCRs of (e) measured by seahorse analysis (n = 4). (g) Cellular TG content of Vector-, WT-P5CS-, and mutant-P5CS-overexpressed AML12 cells followed by treatment with BSA or FFA (n = 5). (h) The P5CS expression level in AML12 cells transfected with Vector, WT-*ALDH18A1* or mutant-*ALDH18A1* plasmids. (i) Cellular TG content of Vector-, WT-P5CS-, and mutant-P5CS-overexpressed primary hepatocytes followed by treatment with BSA or FFA (n = 5). (j) The P5CS expression level in primary hepatocytes transfected with Vector, WT-*ALDH18A1* or mutant-*ALDH18A1* plasmids. Data are shown as mean ± SEM. Two-way ANOVA was used for multi-group comparisons. FFA, free fatty acid; OCR, oxygen consumption rate; PA, palmitic acid; TG, triglyceride.


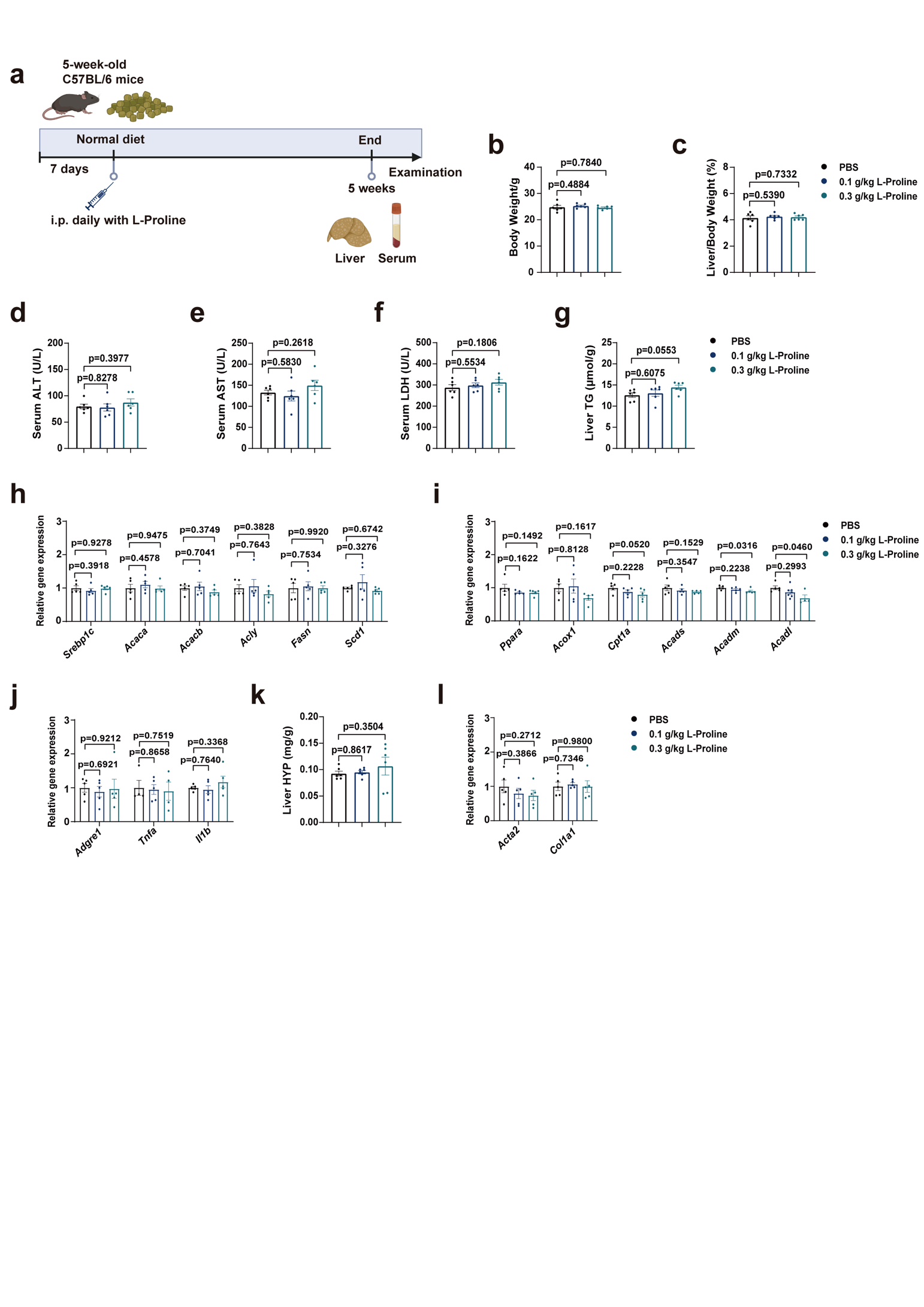


**Supplementary Figure S9. The injection of L-proline has no notable effect on normal mice.**

(a) Mice fed with a normal diet were intraperitoneally injected with L-proline for 5 weeks (n = 6 mice/group). (b) Body weight of mice in the indicated groups (n = 6 mice/group). (c) Liver/Body weight ratio of mice in the indicated groups (n = 6 mice/group). (d-f) Serum ALT (d), AST (e), and LDH (f) levels of the indicated groups of mice (n = 6 mice/group). (g) Hepatic TG content of mice in the indicated groups (n = 6 mice/group). (h) Relative mRNA levels of hepatic fatty acid biosynthesis genes in the indicated groups of mice (n = 4-5 mice/group). (i) Relative mRNA levels of hepatic fatty acid oxidation genes in the indicated groups of mice (n = 4-5 mice/group). (j) Relative mRNA levels of hepatic proinflammatory genes in the indicated groups of mice (n = 4-5 mice/group). (k) Hepatic HYP content of mice in the indicated groups (n = 6 mice/group). (l) Relative mRNA levels of hepatic fibrogenesis-related genes in the indicated groups of mice (n = 4-5 mice/group). Data are shown as mean ± SEM. One-way ANOVA was used for multi-group comparisons. ALT, alanine aminotransferase; AST, aspartate aminotransferase; HYP, hydroxyproline; LDH, lactate dehydrogenase; TG, triglyceride.


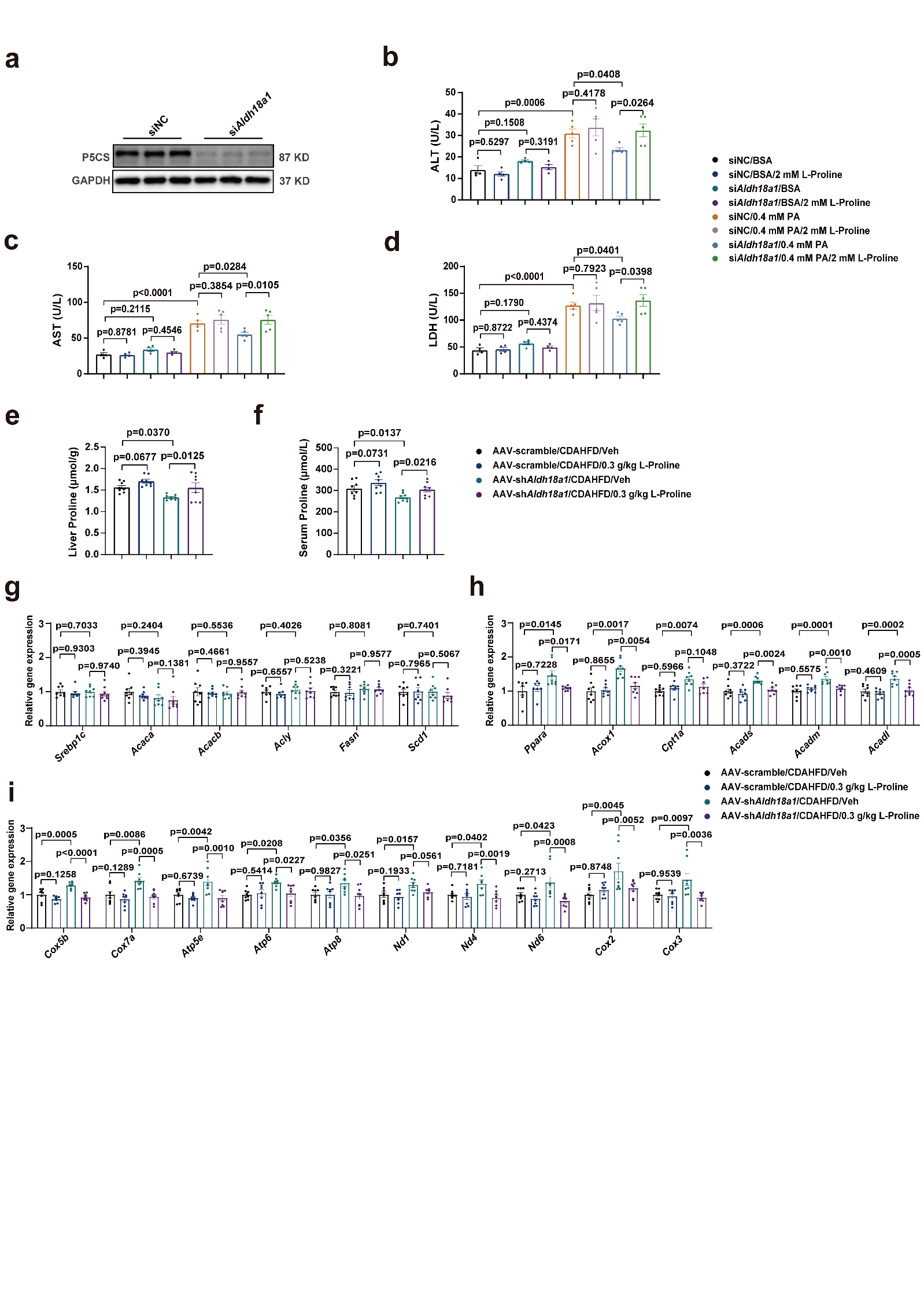


**Supplementary Figure S10. P5CS promotes the progression of MASLD via its downstream enzyme product proline.**

(a) The P5CS expression level in primary hepatocytes transfected with negative control siRNA or si*Aldh18a1*. (b-d) ALT (b), AST (c), and LDH (d) concentrations in the cell supernatants of Negative-control and P5CS-knockdown primary hepatocytes exposed to BSA or PA followed by treatment with or without L-proline (n = 4-5). (e) The liver proline content in the indicated groups of mice (n = 8 mice/group). (f) The serum proline content in the indicated groups of mice (n = 8 mice/group). (g-i) Relative mRNA levels of genes related to fatty acid biosynthesis (g), fatty acid oxidation (h), and mitochondrial function (i) in the livers of mice in the indicated groups (n = 8 mice/group). Data are shown as mean ± SEM. Two-way ANOVA was used for multi-group comparisons. ALT, alanine aminotransferase; AST, aspartate aminotransferase; CDAHFD, choline-deficient, L-amino acid-defined, high-fat diet; LDH, lactate dehydrogenase; PA, palmitic acid.

**Supplementary Table S1: List of antibodies used in the experiments**

| **Antibodies** | **Source** | **Host** | **Dilution** |
| --- | --- | --- | --- |
| anti-GAPDH | Cell Signaling Technology  (#2118) | Rabbit (monoclonal) | 1:10000 (for WB) |
| anti-OAT | Santa Cruz Biotechnology  (#sc-374243) | Mouse (monoclonal) | 1:1000 (for WB) |
| anti-PRODH | Santa Cruz Biotechnology  (#sc-376401) | Mouse (monoclonal) | 1:1000 (for WB) |
| anti-P5CS | Proteintech (#17719-1-AP) | Rabbit (polyclonal) | 1:2000 (for WB)  1:200 (for IHC) |
| anti-P5CDH | Proteintech (#11604-1-AP) | Rabbit (polyclonal) | 1:2000 (for WB) |
| anti-PYCR1 | Proteintech (#13108-1-AP) | Rabbit (polyclonal) | 1:2000 (for WB) |
| anti-PYCR2 | Proteintech (#17146-1-AP) | Rabbit (polyclonal) | 1:2000 (for WB) |
| anti-PYCR3 | ABclonal (#A17763) | Rabbit (polyclonal) | 1:1000 (for WB) |

**Supplementary Table S2: List of primers used in the experiments**

| **QPCR primers** | **Forward primer (5’-3’)** | **Reverse primer (5’-3’)** |
| --- | --- | --- |
| Hs-*GAPDH* | GTCTCCTCTGACTTCAACAGCG | ACCACCCTGTTGCTGTAGCCAA |
| Hs-*ALDH18A1* | GCCCTTCAACCAACATCTTCT | AGGGGTACAGTGATAAACGGG |
| Hs-*CD68* | TGGGGCAGAGCTTCAGTTG | TGGGGCAGGAGAAACTTTGC |
| Hs-*IL1B* | AGCTACGAATCTCCGACCAC | CGTTATCCCATGTGTCGAAGAA |
| Hs-*COL1A1* | AGTCTGTCCTGCGTCCTCTG | TGTTTGGGTCATTTCCACAT |
| Hs-*ACTA2* | CTATGCCTCTGGACGCACAACT | CAGATCCAGACGCATGATGGCA |
| Mm-*Gapdh* | CATCACTGCCACCCAGAAGACTG | ATGCCAGTGAGCTTCCCGTTCAG |
| Mm-*18s rRNA* | AGTCCCTGCCCTTTGTACACA | CGATCCGAGGGCCTCACTA |
| Mm-*Aldh18a1* | GTCCAGCCCTCAGCTATTAGA | CTTCAGCTCACTTCGGTGGG |
| Mm-*Adgre1* | ATGGACAAACCAACTTTCAAGGC | GCAGACTGAGTTAGGACCACAA |
| Mm-*Tnfa* | CTGAACTTCGGGGTGATCGG | GGCTTGTCACTCGAATTTTGAGA |
| Mm-*Il1b* | GCAACTGTTCCTGAACTCAACT | ATCTTTTGGGGTCCGTCAACT |
| Mm-*Tgfb1* | ACCATGCCAACTTCTGTCTGGGAC | ACAACTGCTCCACCTTGGGCTTG |
| Mm-*Acta2* | TGCTGACAGAGGCACCACTGAA | CAGTTGTACGTCCAGAGGCATAG |
| Mm-*Col1a1* | AAGAGGCGAGAGAGGTTTCC | AGAACCATCAGCACCTTTGG |
| Mm-*Srebp1c* | TTGGCCACAGTACCTTTGGTT | CTGAGCCTAGGGCCTTGCT |
| Mm-*Acaca* | ATGGGCGGAATGGTCTCTTTC | TGGGGACCTTGTCTTCATCAT |
| Mm-*Acacb* | CGCTCACCAACAGTAAGGTGG | GCTTGGCAGGGAGTTCCTC |
| Mm-*Acly* | ACCCTTTCACTGGGGATCACA | GACAGGGATCAGGATTTCCTTG |
| Mm-*Fasn* | TGGGTTCTAGCCAGCAGAGT | TACCACCAGAGACCGTTATGC |
| Mm-*Scd1* | TTCTTGCGATACACTCTGGTGC | CGGGATTGAATGTTCTTGTCGT |
| Mm-*Ppara* | TGCAAACTTGGACTTGAACG | GATCAGCATCCCGTCTTTGT |
| Mm-*Acox1* | CTCCCACTCTGGTCTTCCTG | GGTGTAAAAGGTGGCCTGAA |
| Mm-*Cpt1a* | CTCCGCCTGAGCCATGAAG | CACCAGTGATGATGCCATTCT |
| Mm-*Acads* | TGGCGACGGTTACACACTG | GTAGGCCAGGTAATCCAAGCC |
| Mm-*Acadm* | AGCTGCTAGTGGAGCACCAAG | TCGCCATTTCTGCGAGC |
| Mm-*Acadl* | TCACCAACCGTGAAGCTCGA | CCAAAAAGAGGCTAATGCCATG |
| Mm-*Cox5b* | GCTGCATCTGTGAAGAGGACAAC | CAGCTTGTAATGGGTTCCACAGT |
| Mm-*Cox7a* | CAGCGTCATGGTCAGTCTGT | AGAAAACCGTGTGGCAGAGA |
| Mm-*Atp5e* | CAGGCTGGACTCAGCTACATC | GTTCGCTTTGAACTCGGTCTT |
| Mm-*Atp6* | CCTTCAATCCTATTCCCATCC | GTTGGAAAGAATGGAGACGG |
| Mm-*Atp8* | GGCACCTTCACCAAAATCACT | GGGGTAATGAATGAGGCAAATAGA |
| Mm-*Nd1* | CCTAGCCGTTTACTCAATCCT | TGATGGCTAGGGTGACTTCAT |
| Mm-*Nd4* | CTCAGTTAGCCACATAGCA | CCGTTCGTAGTTGGAGTT |
| Mm-*Nd6* | AACTCCAACATCATCAACCT | TGAGGAATATCCAGAGACTTG |
| Mm-*Cox2* | CGTCTGAACTATCCTGCCCG | TGGTAAGGGAGGGATCGTTG |
| Mm-*Cox3* | GCAGGATTCTTCTGAGCGTTCT | GTCAGCAGCCTCCTAGATCATGT |
| Mm-*Pycr1* | ATGAGCGTAGGCTTCATCGG | CGAGACCGTAGCTTGGTCC |
| Mm-*Pycr2* | ATGAGCGTGGGTTTCATCGG | GCTTCGGGTCAGATTCACACC |
| Mm-*Pycr3* | GCACATCATCGTATCTGTGGC | CTCGCAATACTCGTGTGTTCG |
| Mm-*Oat* | GGAGTCCACACCTCAGTCG | CCACATCCCACATATAAATGCCT |
| Mm-*Aldh4a1* | CGATGGAAGCACACCTCTTCT | GGCGACAACTGGTACTGTATATC |
| Mm-*Prodh* | GCACCACGAGCAGTTGTTC | CTTTGTTGTGCCGGATCAGAG |
| **siRNA sequence** | **Sense (5’-3’)** | **Antisense (5’-3’)** |
| Mm-si*Aldh18a1*-#1 | GAUCCUGUUAGCCAACAAATT | UUUGUUGGCUAACAGGAUCTT |
| Mm-si*Aldh18a1*-#2 | CCUGGCAUCUAUUGUUGAATT | UUCAACAAUAGAUGCCAGGTT |
| **Primers for plasmids construction** | **Forward primer (5’-3’)** | **Reverse primer (5’-3’)** |
| pcDNA3.1-*ALDH18A1*-K76A | GAGAATCGTGGTGGCTCTCGGCAGTGCCGTGGTGA | CGAGAGCCACCACGATTCTCTTGGCATGCTTCAGC |
| pcDNA3.1-*ALDH18A1*-D247A | GTGTTAAAGATAATGCTAGCCTGGCTGCCCGACTG | GCTAGCATTATCTTTAACACTAATAACATTTACCCCCTG |
| pcDNA3.1-*ALDH18A1*-K311A | AAGCCGCTGTGAAAGCAGCCCTCTGGGCTTTGC | GGCTGCTTTCACAGCGGCTTCCATGCCACCCATTC |
| pcDNA3.1-*ALDH18A1*-C612A | AGCTGCCGCTAATGCTTTGGAGACTTTGTTAATCC | AAGCATTAGCGGCAGCTGGATATTCACATTTAGA |
